# Offline encoding impaired by epigenetic regulations of monoamines in the guided propagation model of autism

**DOI:** 10.1186/s12868-018-0477-1

**Published:** 2018-12-17

**Authors:** Dominique G. Béroule

**Affiliations:** 1 LIMSI (Computer Sciences Laboratory for Mechanics and Engineering Sciences), CNRS, rue John Von Neumann, Campus Universitaire d’Orsay - Bâtiment 508, 91403 Orsay Cedex, France; 2 CRIIGEN Scientific Council (Committee for Independent Research and Information on Genetic Engineering), 42 rue de Lisbonne, 75008 Paris, France

**Keywords:** Autism spectrum disorders, Computational model, Sex-ratio, Sleep architecture, Memory encoding, Valproic acid, Epigenetics, Serotonin, Monoamine oxidases

## Abstract

**Background:**

Environmental factors can modify the expression of genes, including those involved in the metabolism of neurotransmitters. Accounting for a control role of *monoamine* neurotransmitters, the guided propagation (GP) memory model may contribute to investigate the consequences of neuromodulation impairments on development disorders such as autism. A prenatal transient excess of ‘*monoamine oxidase A’* enzyme is assumed here to trigger persistent epigenetic regulations that would induce imbalanced metabolisms of synaptic monoamines. When imported into the ‘offline’ encoding cycles of a GP model, the consequent ‘serotoninergic noise’ leads to aberrant memory structures that can be linked with autism symptoms.

**Results:**

In computer experiments, different levels of uncoupling between representations of monoamines correlate with the amount of impaired GP modules, the severity of irrelevant connections, as well as network overgrowth. Two types of faulty connections are respectively assumed to underlie autism traits, namely repetitive behavior and perceptual oversensitivity. Besides computational modelling, a genetic family-tree shows how the autism sex-ratio can result from combinations of pharmacological and epigenetic features.

**Conclusions:**

These results suggest that the current rise of autism is favored by three possible sources of biological masking: (1) during sleep, when cyclic variations of monoamines may undergo disrupted enzymatic activities; (2) across generations of ‘healthy carriers’ protected by the X-chromosome silencing and a specific genetic variant; (3) early in life, as long as the brain development draws on pools of neurons born when the transient enzymatic excess and its persistent epigenetic regulation overlapped, and as long as the *B* type of monoamine oxidase does not significantly impact dopamine. A disease-modifying therapy can be derived from this study, which involves relevant biomarkers to be first monitored over several months of clinical trial.

**Electronic supplementary material:**

The online version of this article (10.1186/s12868-018-0477-1) contains supplementary material, which is available to authorized users.

## Background

The highest progression of a spectrum of non-epidemic disorders is observed in countries where chemical substances are consumed the most, including perfumes, detergents and pesticides [1]. With forty times more cases today than in the 60s, and ratio estimates that shifted from 1.7% (in 2010) to 2.4% (*weighted prevalence* [2]) of the US population, these neurodevelopmental disabilities referred to as Autism Spectrum Disorders (ASD) seemingly followed environmental changes. Indeed, among 250 susceptibility genes already analyzed by geneticists, none is involved in more than 2% of ASD cases [3]. However, more than autistic twins who share both genetic and prenatal environmental influences [4], this is the male prevalence (greater than 4:1) that led scientists to suspect a heritable genetic disease implying DNA mutations [5]. However, there is increasing evidence that variations in genes expression caused by the environment can be inherited in humans, with a number of studies reporting transgenerational transmission of these variations without genetic mutations (*epigenetics*) [6].

### Neuromodulators possibly unbalanced by epigenetic regulations

#### Monoamines under influence

Mostly focused on synapses and neurons, genetic investigations have concerned the controlled networking of neurons by neuromodulators, including *serotonin* (5-HT) for its recognized contribution to the brain development [7]. Several studies of autistic troubles have therefore addressed the production, transport and metabolism of 5-HT, depending themselves upon the expression of specific genes. In the blood of autistic patients, high 5-HT levels can be attributed to its increased synthesis by *tryptophan hydroxylase*, enhanced uptake into platelets through transporter, or decreased metabolism by the *monoamine oxidase type A* (MAOA) enzyme [8]. MAOA preferably metabolizes 5-HT among other monoamines, namely *Norepinephrine* (NE) and *Dopamine* (DA). Given its likely coupling with NE, serotonin should be considered in its neuromodulation context. Accordingly, 5-HT and NE variations would tend to accompany each other, except in case of repeated use of drugs of abuse [9]. MAOB, the other type of monoamine oxidase, may also be involved in ASD, since its brain concentrations, barely detectable at birth, reach a high at around 2 years of age [10], when autism symptoms start being revealed.

##### Enzymatic deficiency

In order to avoid synaptic overload, neuromodulators are broken down by enzymes once they have contributed to transmit the nervous signal. The balanced concentration of monoamines in the synaptic cleft thus partly depends on the ongoing enzymatic activity. Compared with control groups, significant reductions of the MAOA enzyme have been recorded in autistic children, either in plasma [11] or in both cerebellum and frontal cortex [12], inducing lower metabolism (degradation, deactivation) of serotonin. The key-role of MAOA is also evidenced in genetically-modified mice with MAO knock-out (KO) gene, displaying autistic features associated with brain abnormalities [13]. Similarly, mutations and deletion in the MAOA gene, as found in the *Brunner Syndrome* (BS) [14], have recently been observed to induce autism symptoms [15]. Essential here as a potential cause of neuromodulation imbalance, the following piece of knowledge must be emphasized: unlike other monoamines, 5-HT cannot be metabolized by another enzyme, namely the *catechol*-*O*-*methyltransferase* (COMT) [16].

##### Genetic variants

Variations in the expression of the MAOA enzyme have been identified which do not involve DNA mutations. Located in the promoter region of genes, *allelic variants* are known as functional polymorphisms, namely *variable number tandem repeat* (uVNTR): the 3-repeat allele is associated with decreased transcriptional activity, whereas the 4-repeat allele shows the opposite effect [17]. Several genes in charge of neuromodulators show allelic variants, including the 5-HT transporter [18] and the COMT enzyme: the *A* (or *Met*) and *G* (or *Val*) alleles respectively exhibit low and high genetic expressions for breaking down the synaptic DA and NE. About 25% of people carry the *AA* (*Met/Met*) polymorphism which brings the lowest metabolism of both DA and NE [19]. Besides a possible synergetic effect with other genetic variants [20], the 3-repeat low-activity *allele* of the MAOA promoter has been linked with cortical enlargement (up to a factor 1.5)—a recognized hallmark of autism [21]—, as well as with ASD severity, namely a tendency towards lower intellectual abilities and more severe behavioral problems [22]. Another specific MAO polymorphism (*rs6323*) with low-activity allele exhibits significant association with ASD, posing higher risk in males [23]. However, despite their involvement in the ASD heterogeneity, these genetic polymorphisms exist independently of the condition, and should not be regarded as its main cause.

##### Epigenetics

The MAOA promoter not only displays built-in variants; it is also sensitive to environmental factors which participate in its *epigenetic* programming. A number of situations have been shown to modify the MAOA expression, including Major Depressive Disorder (MDD) [24], stressful events, diet changes, tobacco smoking, and social environment [25]. At the molecular level, mechanisms have been identified which explain how a *xenobiotic*—foreign chemical substance—can contribute to the acute promotion of MAOA. For instance, *Valproic acid* (VPA) can directly bind to a *transcription factor* of the gene promoter, among other possibilities of action [26]. Quite relevant to the present study, the pre-natal exposure to *xenobiotics* with affinity for endogenous *transcription factors* is likely to induce persistent regulations of genes involved in the enzymatic activity [27]. With respect to interactions between maternal and fetal neuromodulators, exposure to maternal inflammation or MDD imply atypical concentrations of placental 5-HT. This situation may also affect the fetal brain development [28, 29]. Accordingly, both external and internal factors can be involved in the embryonic development of ASD [30].

**Fig. 1:**
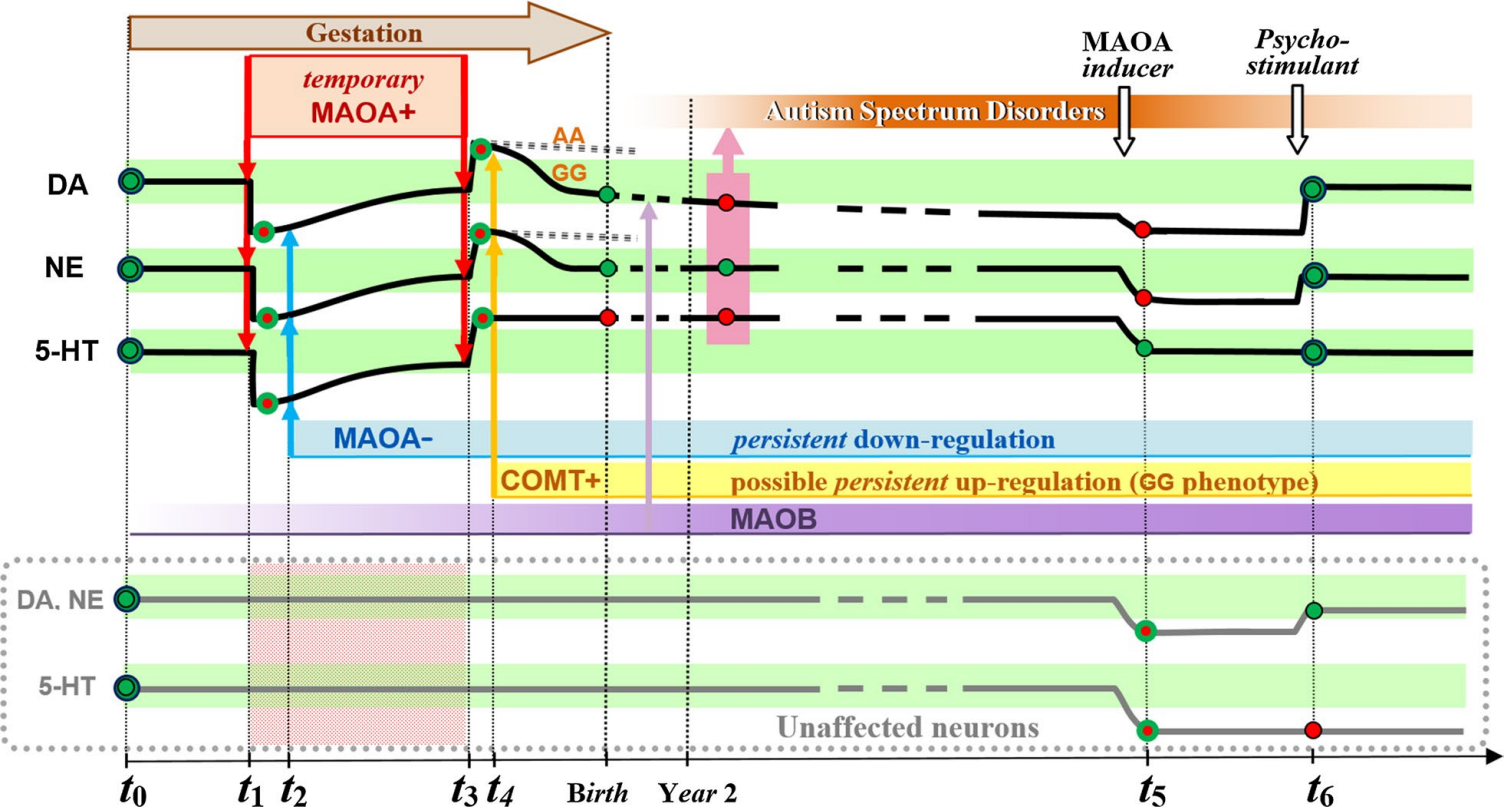
Hypothetical synaptic variations of 3 monoamines average levels (5-HT, DA, NE) in a lineage of neurons the first generations of which undergo a temporary excess of MAOA (labelled here: MAOA+) during gestation. The impact of two drugs intended for restoring balance of these monoamines is displayed on the right-hand side, including in unaffected neurons (lower curves). Red dots show the monoamine levels outside of their core range (green areas). Red dots with a green outline correspond to out-of-range values which however still respect the coupling of 5-HT and NE monoamines. *t*_1_ Beginning of MAOA disruption. *t*_2_ Epigenetic regulation of every monoamine. *t*_3_ End of the disruptive episode, no later than the end of gestation. *t*_4_ Regulation of DA and NE metabolisms by the COMT enzyme as a function of its phenotype (lowest expression with AA, highest with GG). Year 2: The MAOB enzyme is reaching a stage in its rise. Autism symptoms start emerging with a specific pattern of monoamines (indicated by a pink rectangle with an arrow pointing upward to the ASD rise). *t*_5_ Decrease of the 5-HT noise by a MOAO inducer which causes some hyperactivity associated with DA and NE deficits. *t*_6_ Medication of a low-dose psychostimulant against hyperactivity. The two curves at the bottom show the theoretical effect of the treatment in unaffected neurons, eventually resulting in enhanced metabolism of serotonin

Based on the above facts and hypotheses, it is now time to address a main point of the present study. Mutations and promoter variants of the MAOA gene can undoubtedly be inherited. As an alternative, the promoter region of this gene may undergo prenatal acute modifications caused by either xenobiotic intrusion or maternal condition. Among potential xenobiotics, VPA has been found to stimulate the production of MAOA and, consequently, the degradation of 5-HT [26]. Unexpectedly, if taken during pregnancy, this small molecule is known to possibly generate autistic features in the offspring [31], which have been linked with high 5-HT synaptic levels, instead. This apparent contradiction can be resolved by discriminating two separate contexts in which VPA may reach the brain. The first one is during sensitive periods of the prenatal development, when genetic programming can still counteract a temporary acute imbalance of monoamines (MAOA+ in Fig. 1) [32]. As a small molecule with two *methyl groups* (CH3), VPA can cross the fetus blood-brain barrier and bind to DNA transcription factors (e.g.: *Sp1* [26]) together with endogenous proteins. Among several epigenetic processes that are able to fight back against the resulting overexpression of MAOA, protein *R1* is known to act through Sp1 sites as well [33]. Now, regarding the postnatal context, monoamine metabolisms may then have been permanently programmed through reactions to an initial contact with VPA. Although still inducing MAOA, VPA would not trigger the same epigenetic regulation as in the prenatal context, namely within critical periods of the fetal brain development. The scenario proposed in Fig. 1 shows how a coincident under-metabolism of 5-HT and over-metabolism of DA can result from the meeting of epigenetic marks with an allelic variant. Following a temporary xenobiotic intrusion (e.g.: VPA), a MAOA down-regulation allows monoamines to return to their baseline value. But when the temporary/accidental increase of MAOA is over, monoamines are less metabolized again, hence a significant increase of their average level. Both DA and NE under-metabolisms can then be compensated by another enzyme (COMT) before MAOB gradually takes part in DA degradation, after birth. This scenario could explain the relative excess of synaptic 5-HT observed long after the prenatal intake of a drug which paradoxically tends to clear the synapse from 5-HT, among other monoamines.

Now, there is a connection waiting to be made between these early molecular events and the late onset of autistic conditions. If a theory could bridge the gap between a prenatal molecular accident and postnatal behavioral deficiencies, it should also account for the delayed occurrence of symptoms, several years after birth in children who manifest developmental regression [34]. Among relevant factors to be specified hereafter, low MAOB levels during gestation could mask the uncoupling of NE and 5-HT metabolisms which is assumed here to underlie ASD (Fig. 1).

### Computer simulations of impaired encoding

Reactions of the nervous system against the poor degradation of 5-HT at issue may include the reduction of serotonin receptors evidenced in limbic and neocortical regions of the autistic brain [35]. Along the same line, anatomical studies reveal reciprocal connections between the 5-HT generator (*raphe nuclei*) and its cortical targets, allowing a feedback control of cortical 5-HT release [36]. Exercised ‘online’ in the awake brain, this control loop is likely to compensate for excess 5-HT, together with the reuptake mechanism which is known to contribute to 5-HT synaptic clearance. However, an enzymatic deficit cannot be counteracted when the release of 5-HT is interrupted whatever the cortical feedback, as across sleep cycles: there, 5-HT neurons decrease their firing and stop activity during the last phase of each cycle [37]. Consequently, if this interruption fulfilled a key function during sleep, the faulty persistence of monoamines in the synaptic cleft could not be counteracted by ‘online’ mechanisms. Such irrelevant synaptic accumulation of serotonin during sleep cycles is central in the theory proposed here.

#### Sleep and parallel processing

From a broader perspective, the medical descriptions of ASD have evolved over last decades. After having enhanced social deficits, they shifted towards communication problems and repetitive/restricted behaviors [38]. Today, sleep problems are also increasingly reported. Whereas healthy adults sleep across 4 or 5 cycles of distinct phases, autistic children are observed to hardly fall asleep, sleep a few, with delayed phases and significantly less ‘deep-sleep’. Although identified in animal models as well [39], these specific troubles are surprisingly not mentioned among the official criteria of autism [38]: sleep disorders are rather thought to originate in the daytime over-activity and stress [40], only worsening the condition disabilities [41].

The lifelong alternation of wake and sleep involves memory processes that are respectively referred to as encoding, consolidation and recall. Sleep is widely thought to contribute to the consolidation stage by strengthening fragile memories [42], then available on the long-term for effective recall. Such consolidation during sleep may accompany the transfer of information between the *hippocampus* and cortical areas [43], and sleep phases can respectively be associated with distinct types of memory [44]. Contrary to waking in which brain areas are busy interacting, sleep would allow the independent consolidation of memories in different neural structures [45]. During *Slow*-*Wave*-*Sleep* (SWS), the hippocampus is evidenced to form memories of events in time [46], and the ‘two-stage memory’ system postulates a role of short-term recipient for information to be distributed in cortical areas [47]. In the final *Rapid Eye Movement* (REM) phase of a sleep cycle, the brain paradoxically shows greater activity than during wake, whereas the body remains still. Across these distinct phases, a saw-teeth neuromodulation pattern makes the production of both 5-HT and NE monoamines decline down to zero from the beginning of each cycle until REM sleep [48], while DA levels are maintained.

Whatever the model, a division of labor is currently supported whereby the part of sleep would pertain to the strengthening of memories encoded during prior wakefulness [42–47]. Although few structural modifications of the neural tissue have been observed during sleep so far (i.e.: the expansion of synaptic boutons in *drosophila* [49]), it is argued here that offline periods could also take part in memory encoding. Indeed, with evidence of shortly delayed activities in distant neural networks (e.g.: hippocampus and neocortex [43, 50]) during sleep phases, it appears feasible for brain areas to encode inner stimuli while the latter are generated by the hippocampus. Sleep would thus contribute significantly to the proper development of the brain, a function vital enough to justify that one third of the human life is spent asleep, albeit in a competitive environment.

#### Genesis of a learning constraint

When initiated in 1983, the Guided Propagation (GP) system implemented concept-cells named *Event*-*Detectors* (*EDs*), considered as potential targets for *inner*-*flows* of activity. The other main feature of this deterministic approach was the recruitment of new cells for growing the *memory paths* ending in EDs, in response to requests of a dynamic learning algorithm [51]. In this formalism, the direction/path taken by a given inner-flow depends on the occurrence of afferent stimuli, one after the other, hence the expression: “guided propagation”. Within module *k*, the inner-flow is initiated by a *root*-*cell*, and flows along the parallel paths chains of GP-cells (formerly named *Elementary Processing Units*) coding for sequences of events (e.g.: [*root*-*cell*_*k*0_] => [*cell*_*k*1_:‘M’] => [*cell*_*k*2_:‘A’] => [*cell*_*k*3_:‘O’] => [*cell*_*k*4_:‘B’] => *concept*-*cell*_*k*5_: ‘MAOB’). In order to control the necessary balance between inner (contextual) flows and stimuli, only two control parameters are associated with every cell, namely the ratio *R* between the respective weights of Context (C) and Stimulus (S) inputs, and *Excitability E* which defines the cell response threshold [52]. Surprisingly, this minimalist design could later on be used to model coupling between 5-HT and NE (through *R*), as well as a hypothetical global function of DA neuromodulation (through *E*) [53]. Definitions of the GP system components are given in Table 1, while a video movie shows a glimpse of running computer simulation [54].

**Table 1:** Synthetic description of a guided propagation system

Elements of guided-propagation (GP)	Definitions
GP architecture	Prewired set of processing modules defined by their relative connections within a matrix of channels and layers. The GP software deals with the modulation and growth from scratch of every module, thus simulating a global control device. Banks of elementary detectors and effectors form the predefined input/output of the full architecture
GP-cells Elementary processing units (EPU)	Network nodes within every module. GP-cells are chained for encoding sequences of stimuli, giving rise to a dynamic tree-like structure that receives either stimuli or modulation from other modules. At least three types of GP-cells can be distinguished: the root unit, Context-Dependent units, and Event Detectors/Generators
Context-dependent (CD) unit	Main GP-cell, possibly driven by a contextual input from its upstream predecessor along a tree branch. The other activating input is a stimulus generated by the output of another module. A CD unit responds to the possible coincidence between its Contextual (**C**) and Stimulus (**S**) inputs
Event detector/generator (ED/G)	GP-cell located at the end of a module branch, among outputs which interface with other modules
Memory path	Chained GP-cells, from a module root to an ED/G
GP-cell excitability (*E*) and response threshold	The Excitability parameter E_*i*_ defines the total input required for cell *i* to fire, with a response threshold [**S**(t − τ_*ij*_) + **C**(t − τ_*ik*_)]/E_*i*_, where τ_*ij*_ codes for the time-delay of cell *i* input connection from cell *j*
Ratio R between the two GP-cell inputs	Combined with E, the R parameter of a GP-cell allows its operating mode to be dynamically changed
GP-cell operating modes	Different behaviors of a given cell (Fig. 4) are dynamically set by both computer program and running modules:
1. Free	1. Uncontrolled response (to be avoided)
2. Inhibited	2. No response
3. Stimulus-driven	3. Can respond irrelevant of the ongoing context (to be used with caution)
4. Context-driven	4. Can be fully activated by the only inner-flow (“Anticipation” mode)
5. Restricted	5. Requires both input to possibly respond (“quite”/Learning mode)
Dynamic learning algorithm	A module set in the relevant (“Restricted”) operating mode allows the encoding of unexpected stimuli
a. Differentiation	a. Sprouting of a new branch/’memory path’, provided free GP-cells.
b. Generalization	b. Connection of a stimulus (ED/EG) with a memory path (CD unit).

GP networks could hardly be considered as neurobiological models at the time they were first issued. Altogether, concept-cells in a self-growing network did not meet the notion of “distributed memory” in fashion at that time, neither the dogma stating a fixed number of neurons at birth. Since then, concept-cells have regained interest with the discovery of neurons that respond selectively to either high-level notion [55] or action [56]. As well, the genesis of neurons across a lifetime became plausible with the relation found between song learning and the production of new neurons in a *vocal nucleus* of the adult canary brain [57]. More recently, a strong adult neurogenesis has even been evidenced in the human hippocampus [58]. Provided environmental enrichment, experimental results in adult *guinea pigs* further suggested that new neurons are generated in the cortex of mammals [59]. In a functional perspective, newborn neurons ensure the formation of non-overlapping representations for distinct patterns, whereas older cells mediate pattern completion [60], a biological hypothesis consistent with the *Differentiation*/*Generalization* alternative implemented in GP-networks (Fig. 10 of the “Methods” section).

The first implementation of GP-based model concerned reading [61], and enabled an additional functionality. Memory paths built for perceiving words were found to possibly produce them, through offsets of propagation thresholds. The expression “guided propagation” found a new meaning then: whereas a given series of stimuli could guide an inner-flow forwardly towards a specific ED, modulation signals targeting this ED could aspirate the upstream inner-flow and “generate” the same sequence of stimuli. It thus appeared that an ED could be used as Event Generator (EG). This dual function evokes *mirror neurons*, firing either for the perception of someone acting or for the production of the same action [62]. Action production with GP could later on be applied to the modelling of four symptoms of the *Parkinson disease* [53]. This study originated from the assumption that a local lack of dopamine could functionally compare to a GP-cell high *Excitability*. Not only this parameter, but also the dynamic weighting of GP flows could be linked with the reciprocal control, or coupling, between noradrenergic and serotonergic neurons [63]. Based on the same simplified representation of neuromodulation, a computer implementation of decision-making has more recently been designed, in line with the *ascending spiral* neurobiological model [64]. For this purpose, parallel GP-channels can be connected through cross-circuits aimed at propagating modulating signals from “emotional” to “conditioner” and eventually “sensorimotor” channels where decisions are thus quickly facilitated/repressed [65, 66]. But this anticipation skill brought an inescapable constraint of the original learning algorithm to the surface.

**Fig. 2:**
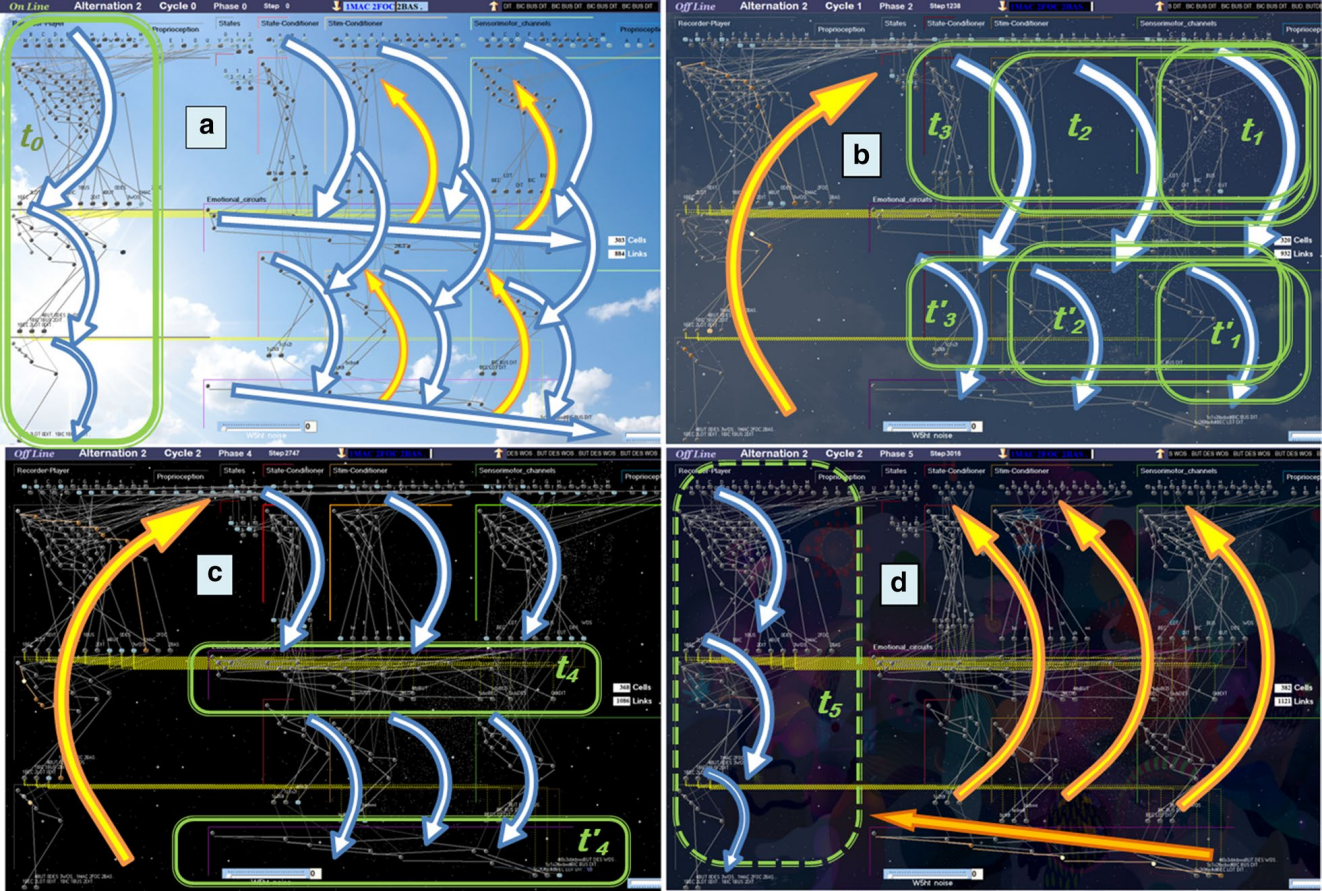
Flows of activation (downward blue arrows) and facilitation (upward yellow arrows) during an alternation of ‘online’ (**a**) and ‘offline’ phases (**b–d**). A video screen capture of the full process can be watched online [54]. Boxes in green surround the GP-modules that are busy encoding. **a** The left-most channel (*Hippo*) records online events, while other channels mainly run in the “Context-driven” anticipation mode. **b** Phases 1–3: channels 3, 2 and 1 gradually join the encoding of events replayed offline by *Hippo*. **c** Phase 4: cross-circuits are grown for linking the output of paths that have just been created in parallel channels. **d** Phase 5: replay of every channel from the deepest cross-circuits (at the bottom-right)

The GP ability to encode stimuli sequences actually relies on their synchrony with the host-module inner-flow. As long as these two flows run in pace, a “never-ending learning” algorithm can apply to various tasks [67]; but the picture changes when unconditioned stimuli have to be anticipated for making decisions, implying that the internal representations of future possible events are activated in advance. By boosting their inner-flows, the forward-looking emotional/conditioner channels can run ahead of the current input. In the context of lifelong learning, the challenge is thus to deal with the implementation of two skills, anticipation and encoding, which share the same memory space but require incompatible regulations (different values of the same parameters). If a learning episode were triggered during anticipation, even known patterns would hardly match their internal references. This lack of coincidence being the criteria for the system to upgrade its knowledge, the network would tend to grow too much. Furthermore, the system proactivity would forbid the accurate sprouting of new memory-paths for implementing *Differentiation*, while favoring the over-*Generalization* of existing paths by several stimuli. A solution to this problem was inspired by the waking/sleep alternation. Proactivity can actually be shut down across ‘offline’ periods during which memory is disconnected from external stimuli, making anticipation temporarily useless. A new channel (named *Hippo* in the following) is required for the online “recording” of online events to be replayed offline and possibly encoded in relevant parallel channels, including their cross-connections (Fig. 2). Of note, these *Hippo* replays appear especially useful for the long-term encoding of behaviors that cannot be rehearsed online. On the biological side, while the overnight reinforcement of neural structures still remains a controversial issue [68], the stronger assumption of a possible involvement of sleep in neural encoding had seemingly not yet been made, so far.

#### Offline cycles of memory encoding: a new model

**Fig. 3:**
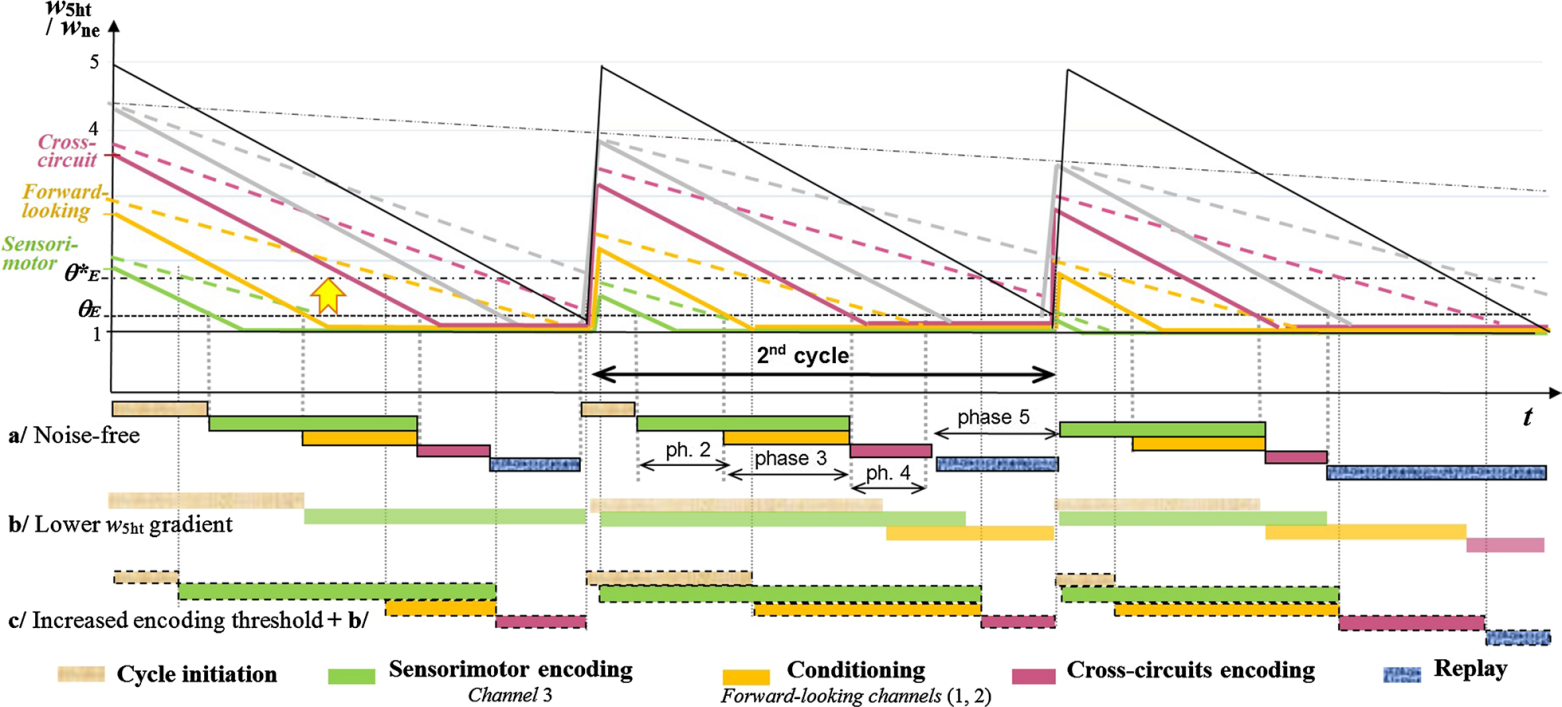
Saw-teeth variation of *R* = *w*_5ht_/*w*_ne_ in GP-modules across offline cycles and phases. To the bottom: corresponding time-course of encoding in memory channels, either noise-free (a/) or with *w*_5ht_ noise (b/, c/). Dotted items represent the same profile modified by a lower *R* gradient standing for the *w*_5ht_ noise, once the encoding threshold *θ*_*E*_ has been set up to θ*_E_ in order to better approximate the noise-free situation

As previously stated, sleep disorders are commonly believed to originate in the daytime over-activity and stress generated by the autistic condition. The opposite causal relationship is supported here. Provided a new interpretation of the sleep architecture (Fig. 3), impaired sleep would disturb the completion of connections within and between parallel neural structures. The resulting miscoded internal representations, as well as their cross-links, would impede several functions relating Perception, Emotion and Language. In fact, these functions are more or less impacted across the autism spectrum. In regular perception, the eyes saccades rely on peripheral vision for the central/foveal vision to run properly [69]. Similarly in everyday decision-making, sensorimotor circuits can instantaneously be driven by emotional channels through an *ascending spiral* pathway [64], while language processing requires at least semantic and syntactic representations to interact. For these various skills, the GP parallel architecture suggests that approximate-and-fast channels guide quick decisions made by accurate-and-slow channels. In autism, neural counterparts of GP processing channels and their cross-links may not properly develop. Indeed, the more severe the injury, the worse an autistic individual performs at tasks requiring parallel and interacting processes, such as decision-making based on emotional conditioning, visual tracking, and the production of speech enriched by prosody.

**Fig. 4:**
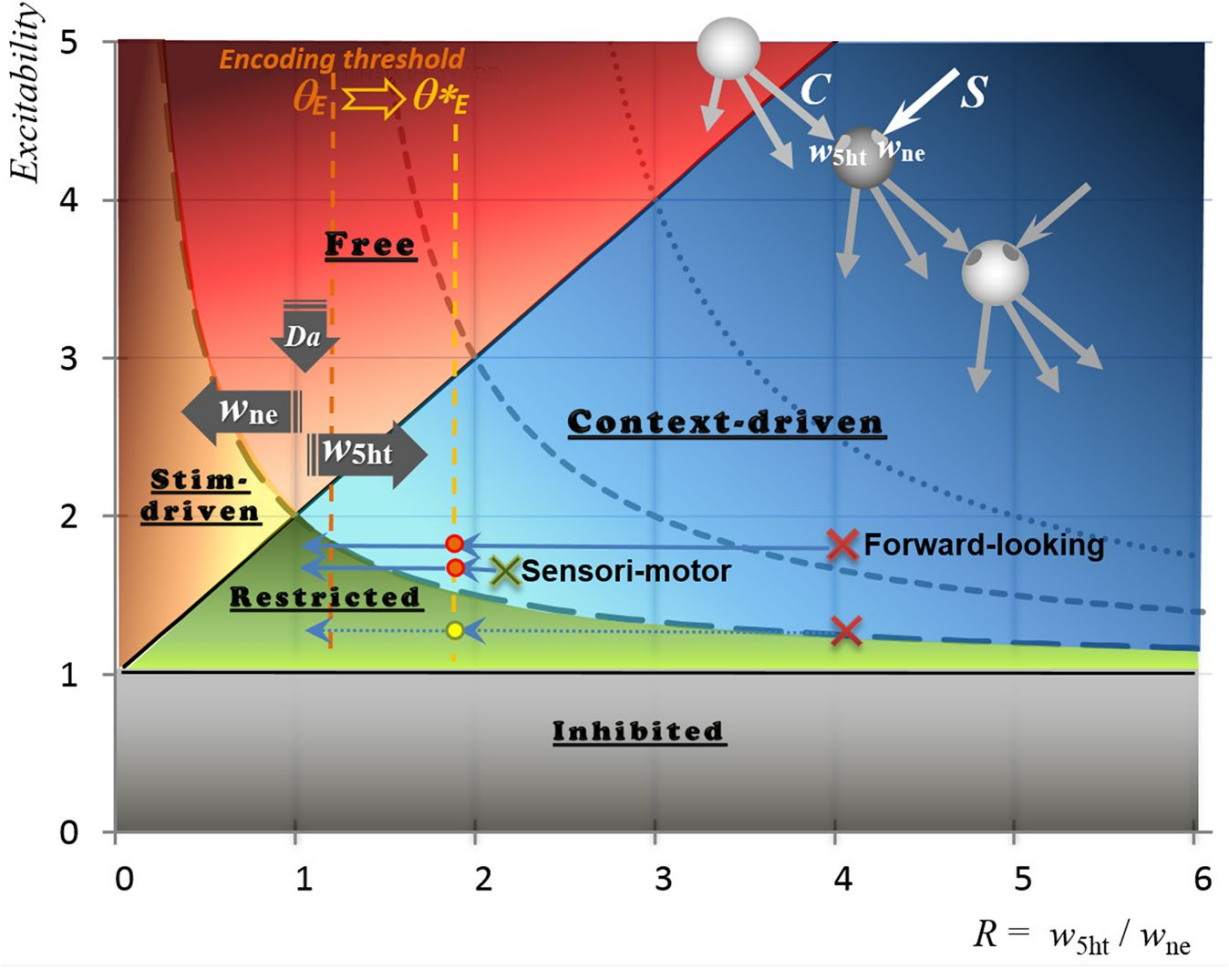
Modulation of GP-cell based on two control parameters. As shown to the upper right, cells are chained for integrating series of stimuli. Each cell can be driven by a Contextual (**C**) input from its predecessor in the chain, and can also be activated by a stimulus (**S**). Depending on its *Excitability* (vertical axis) and the ratio *R* between its contextual weight *w*_5ht_ and its stimulus weight *w*_ne_ (horizontal axis), a given cell runs in one of five operating modes: ***Inhibited***, ***Restricted***, ***Context*-*driven***, ***Stimulus*-*driven***, and ***Free***. The right-most red crosses stand for cells of forward-looking channels, implementing strong anticipation in the *context*-*driven* area. The blue left arrows reflect the *R* decrease during an offline cycle (Fig. 3), towards the *restricted* mode that ensures proper growth of memory paths. However, this encoding process may start as soon as the threshold *θ*_*E*_ is reached. In case of *w*_5ht_ noise, *θ*_*E*_ can be set up to θ*_E_ in order to restore the proper sequence of offline phases, at the expense of the connectivity. The red dots indicate (*R*, *E*) values that entail impaired encoding; the yellow dot meets proper encoding, provided low *E* (high ***Da***) towards the bottom of the “*Restricted”* area. This setting of GP parameters is consistent with the pattern of monoamines assumed in Fig. 1 to underlie autism, namely low 5-HT and high DA metabolisms

The decrease of *R* (*w*_5ht_/*w*_ne_) displayed in both Figs. 3 and 4 represents 5-HT/NE during a sleep cycle. The regular timing of offline phases within each cycle results from the maximum value of *R* at the beginning of each cycle: lowest in sensorimotor modules, medium in forward-looking modules, and highest in cross-circuits. This timing is disturbed when *R* does not decrease fast enough, which is a way to model a MAOA deficiency entailing an insufficient degradation of 5-HT.

## Results

This study consists of three parts that are summarized in Table 2, while more details on the computer simulation are provided in the “Methods” section.

**Table 2:**
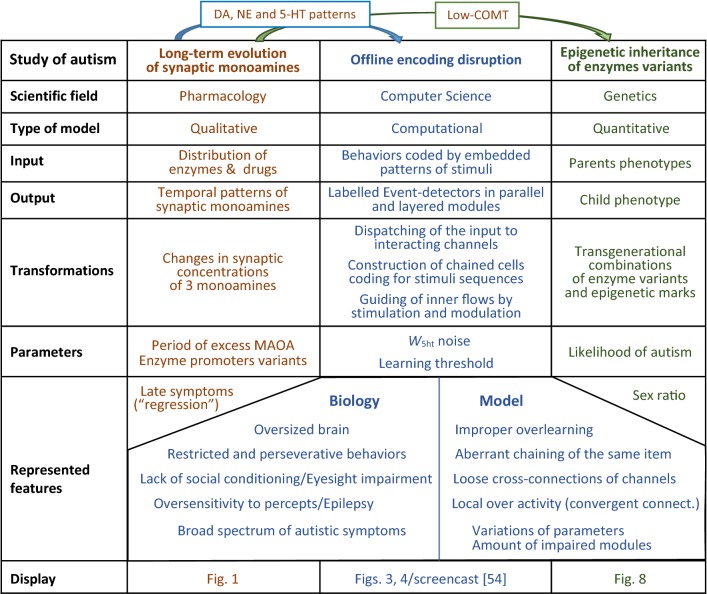
Components of the GP model

In computer simulations of this model, symbolic inputs/outputs standing for behaviors were first used to grow a baseline GP-network across simulated online/offline alternations. This template could then serve as a reference network to be compared to other networks obtained with the same set of inputs/outputs but different levels of *w*_5ht_ noise.

### Dysregulated modulation induces irrelevant connections

Within the memory model introduced above, offline encoding completes the acquisition of emotional/conditioned/sensorimotor patterns representing behaviors, in particular those that cannot be rehearsed online. Each GP module is allowed to extend its representations as soon as the *w*_5ht_*/w*_ne_ ratio of its cells is small enough. But what would happen in case of excess *w*_5ht_ when encoding is permitted within a given module?

**Fig. 5:**
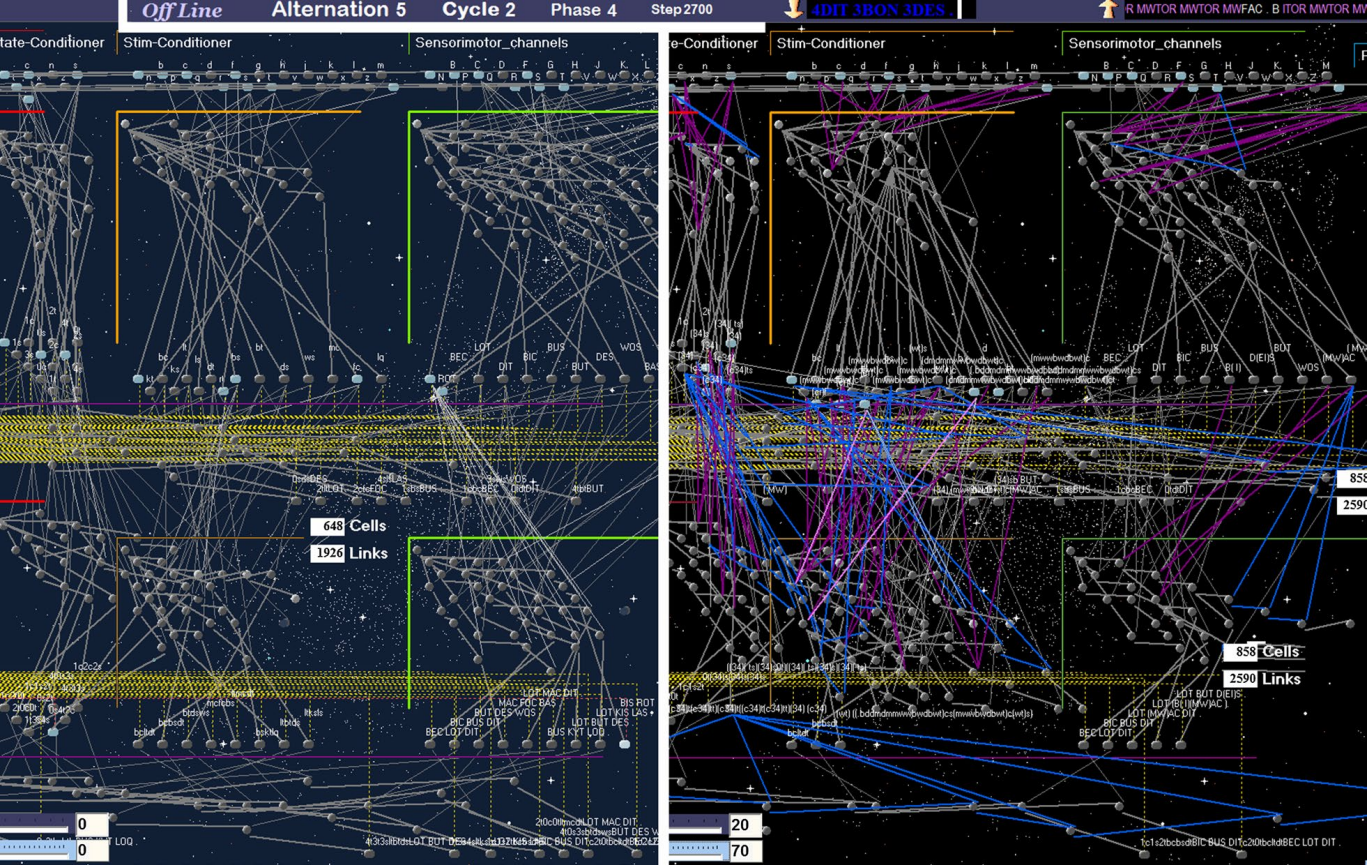
GP computer simulations either noise-free (on the left side) or with *w*_5ht_ noise (on the right). Two screenshots captured at the same processing clock (displayed at the top) show the respective GP networks fed with the same set of input during 5 online/offline alternations. Compared to regular memory paths, two types of structural aberrations appear in the ‘noisy’ network: 1/ convergence of many stimuli towards the same location (purple links), and 2/ divergence of a single elementary pattern towards the same memory-path (in blue). Shortly after the beginning of a 50 alternations trial, the ‘pathological’ network is already 1.3 times bigger than the reference one when comparing their respective Cells and Links counters

**Fig. 6:**
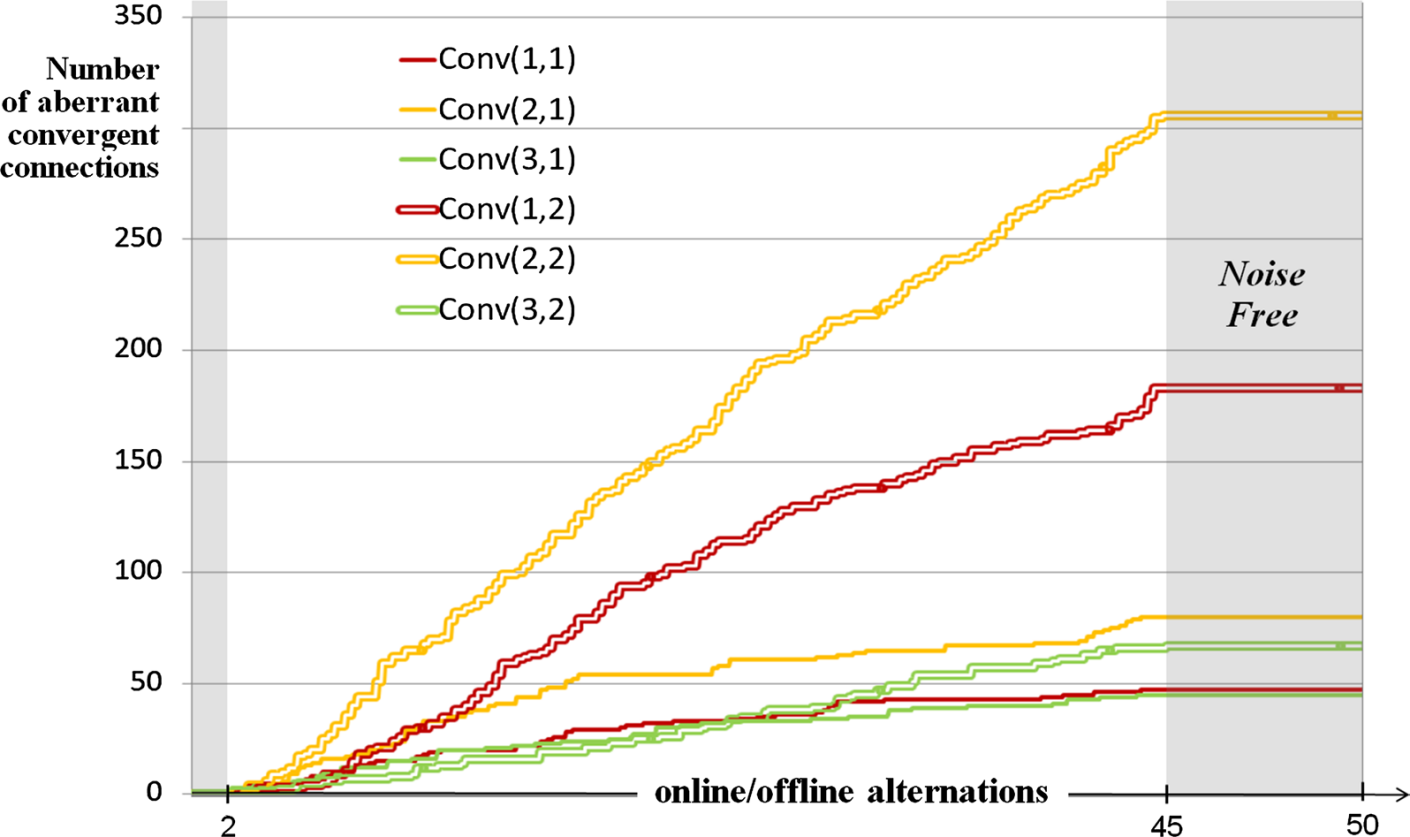
Curves showing the rising of aberrant structures over 50 online/offline alternations. Faulty memory paths stop growing during noise-free periods (in gray). For a given noise level (θ*_E_ = 1.6, here), modules of the 2nd level (double lines) which integrate compound patterns, as well as the forward-looking channels (in yellow and red), are significantly more impacted by the *w*_5ht_ noise than the sensorimotor channel (in green)

**Fig. 7:**
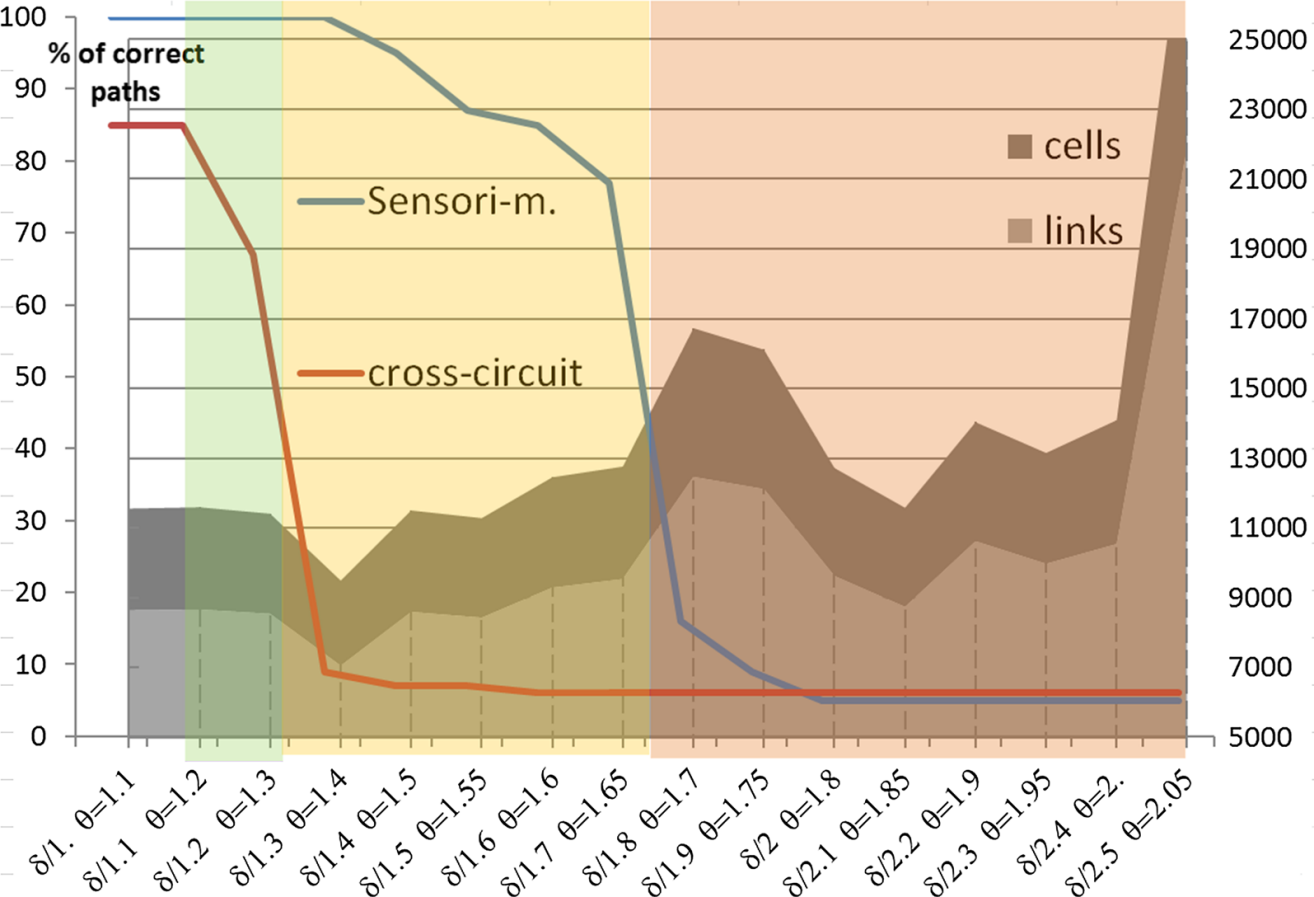
Amounts of cells, links (right-hand axis), and correct paths (left-hand axis) in GP-networks, as a function of 16 different settings (horizontal axis). Each network results from 50 online/offline alternations with a noise ranging along the horizontal axis from *D*_5ht_ = 1 to 2.5 (divider of the *w*_5ht_ gradient δ). Red and blue curves give the rate of intact memory paths classified in three intervals. Impairment level 1 (translucent green area): only cross-circuits are slightly altered. Level 2 (translucent yellow area): the cross-circuit is significantly altered, contains fewer effective paths, and the sensorimotor channel starts hosting aberrant paths. Level 3 (translucent red area): both types of paths are impaired and networks grow significantly above their noise-free size

**Fig. 8:**
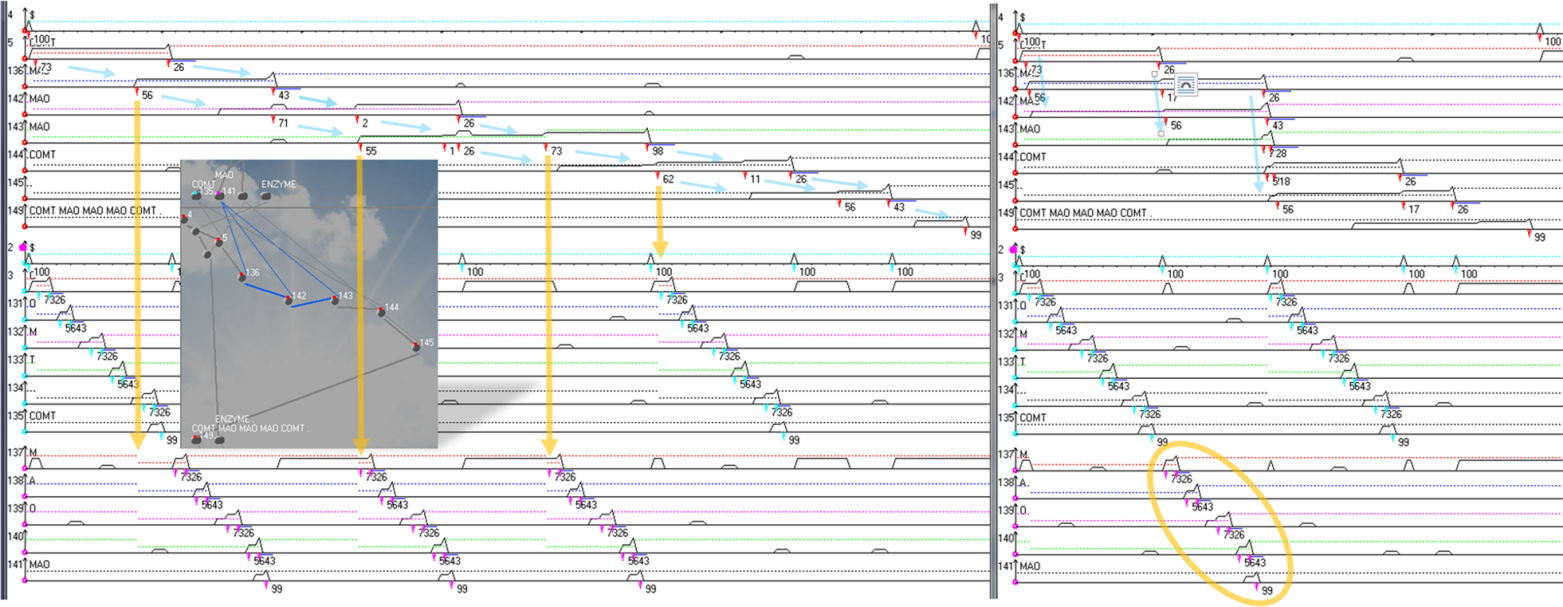
Recovery—through reduction of time-delays—of a pattern generated by its faulty memory path. Aberrant links are displayed in blue on a screenshot of a 2nd-layer memory path labelled “COMT MAO MAO MAO COMT”, formerly built from the sequence “COMT MAO COMT”. Consequently, activity histograms on the left side show how the “MAO” 1^st^-layer path is activated three time (graphs at the bottom). By contrast, the same path generates only one “MAO” occurrence on the right side (circled pattern of activity) when the delayed propagation between chained GP-cells (light blue arrows) have been suspended (temporarily set to zero). The yellow arrows indicate the causal relationship between responses of the 2^nd^-layer path and threshold offsets that generate activity propagating along 1^st^-layer paths (at the bottom)

In computer experiments, a poor degradation of 5-HT is modelled by a slower decrease of the baseline *w*_5ht_*/w*_ne_ gradient. For this purpose, *D*_5ht_ is brought into play as the parameter that divides the regular gradient. In Fig. 3, the higher *D*_5ht_, the more delayed the onset of each phase; the *5*^*th*^ *phase* can even be skipped, similarly to REM-sleep in autism [41]. The phases integrity can however be preserved by raising the encoding threshold (from *θ*_*E*_ to θ*_E_ in Figs. 3 and 4). But due to this compensatory mechanism, encoding is more likely to be run in the *context*-*driven* rather than the *restricted* area, thus possibly altering the network connectivity (Fig. 5). When driven by the inner contextual flow, a given module anticipates next events by activating relevant GP-cells in advance. Because of the consequent lack of coincidence between C and S inputs, even known incoming stimuli cannot match their internal references, which is the criteria for the GP learning algorithm to come into play (Fig. 10 of the “Methods” section), hence GP network overgrowth. Figure 6 shows the near-asymptotic expansion of aberrant structures during 50 online/offline alternations, for a given *w*_5ht_ noise (constant *D*_5ht_ and θ*_E_). The final network size varies in proportion to *D*_5ht_ and θ*_E_. Compared to its noise-free reference, it is enlarged by up to 50% for *D*_5ht_ ranging from 1.8 to 2 (Fig. 7). Although of surprising intensity, this overgrowth appears to be consistent with recent neurobiological findings [21, 70, 71]. Indeed, if inappropriately called in the context-driven area, the encoding process generates both overgeneralization and faulty differentiation. Generalization only creates new connections: many stimuli converging towards a single memory location eventually favor the over-activity as well as the over-generalization of existing memory references, which may be linked with perceptual impairments in ASD, including sensory hypersensitivity. For its part, *Differentiation* implements new GP-cells as well as new connections for building memory paths fed by incoming stimuli. Within the hierarchical structure of GP networks, these stimuli form the output of more peripheral memory paths. Although locked to the rate of its incoming stimuli in the *restricted* mode, such path spreads successive waves of activity in the *context*-*driven* mode, thus faking repeats of the same pattern. If encoded by a deeper module, the consequent repetitive activity is mistakenly encoded as a sequence of the same stimulus (blue links in Figs. 5 and 8). When used online as pattern generators (Fig. 8) these memory paths generate irrelevant repetitions of elementary patterns, including miscoded ones: a possible representation of *stereotyping.*

Additionally, the forward-looking channels are more sensitive to excess *w*_5ht_ than the sensorimotor one, which leads to three levels of global impairment (Fig. 7). Above *D*_5ht_ = 2 and θ*_E_ = 1.8, only the first GP-paths created before the noise onset remain safe, together with all the *Hippo* paths that are supposed not to undergo any disruption in this model. In the network resulting from a gradient only 10% lower than its noise-free value, impairments are limited to the cross-circuits that convey emotional facilitation/repression towards the sensorimotor—decision making—channel. The 2nd and 3rd impairment levels begin for ‘decreases in descent’ which respectively represent 20% and 40% of the baseline gradient.

While online, using the *Hippo* memory rather than impaired channels could be put forward as a solution for overcoming dysfunctions. *Hippo* however misses the parallel processing offered by the full system (e.g.: complementary interacting channels, simultaneous perceptions and productions, emotional circuits). As a positive feature, it should be noted that irrelevant structures are not built anymore as soon as the *w*_5ht_ noise is reset (Fig. 6). Furthermore, although aberrant internal representations are not erased, their online access can be repressed through counterconditioning [66]. However, new branches can still sprout from existing over-generalized paths, unless events occur in a context different from all those previously experienced with a *w*_5ht_ noise.

### Overcome of a structural defect through modulation

According to the GP-model, these are structural impairments generated by monoamines metabolism disruptions that would mediate ASD. In the prospect of dealing with related behavioral deficits, an extra computer simulation focused on a specific type of connectivity assumed here to underpin stereotypy. Although not yet introduced, temporal parameters are key for GP-cell inputs to be able to coincide and also be robust to stimuli time-variability [52]. Although initially set during the sprouting of a new memory path, these parameters associated with every cell input can be modulated so as to dynamically change the pace of pattern generation. As displayed in Fig. 8, an acute reduction of ‘contextual input’ time delays could avoid faulty repetitions of the same item.

### Linking the autism sex-ratio with masking phenomena

So far, the fact that boys are four times more likely to develop ASD than are girls remains largely unexplained. Since genetic combinations are necessarily involved in this prevalence, breeding must find its way despite the poor social skills that characterize the condition. In women, one knows that the existence of two copies of the X chromosome requires the inactivation of one copy in each host-cell in order to avoid the production of twice more proteins than in men. In case of disrupted promotion of gene (e.g.: MAOA) carried by X, this epigenetic regulation—known as ‘chromosome silencing’—allows the safe-X cells to compensate for the other ones, like in hemophilia “healthy carriers”. Similarly, a mosaic [72] of two X-related populations of neurons may avoid an autistic development when one population is impaired, unless both X have been altered. Since they possess a single X chromosome, another masking effect should be found in men. This is why a genetic factor is proposed here to participate in the genesis of ASD, namely the low-COMT phenotype. Rather than the weight of each cell input, the GP theory considers the *w*_5ht_*/w*_ne_ ratio parameter as significant, thus implementing a formal coupling between *w*_5ht_ and *w*_ne_. Consequently, an excessive *w*_5ht_ can be hidden by an increased *w*_ne_. In biological words, a high 5-HT level could be compensated by a supplement of NE. While the metabolism of 5-HT only depends of MAOA, NE can also be degraded by the COMT enzyme. Interestingly here, the low-activity allele of the COMT gene induces a low degradation of NE in 25% of individuals, those who are homozygous for this polymorphism variant [13]. Thus, assuming the 5HT–NE coupling, an epigenetic down-regulation of MAOA could go unnoticed in a population of low-COMT, provided the inheritance of such gene programming. Especially if maintained by chronic exposure to xenobiotics, this epigenetic regulation may cross generations, as already acknowledged in plants. Although still questioned in mammals [73], the transgenerational inheritance of epigenetic traits may contribute to a theoretical explanation of the autism sex-ratio.

**Fig. 9:**
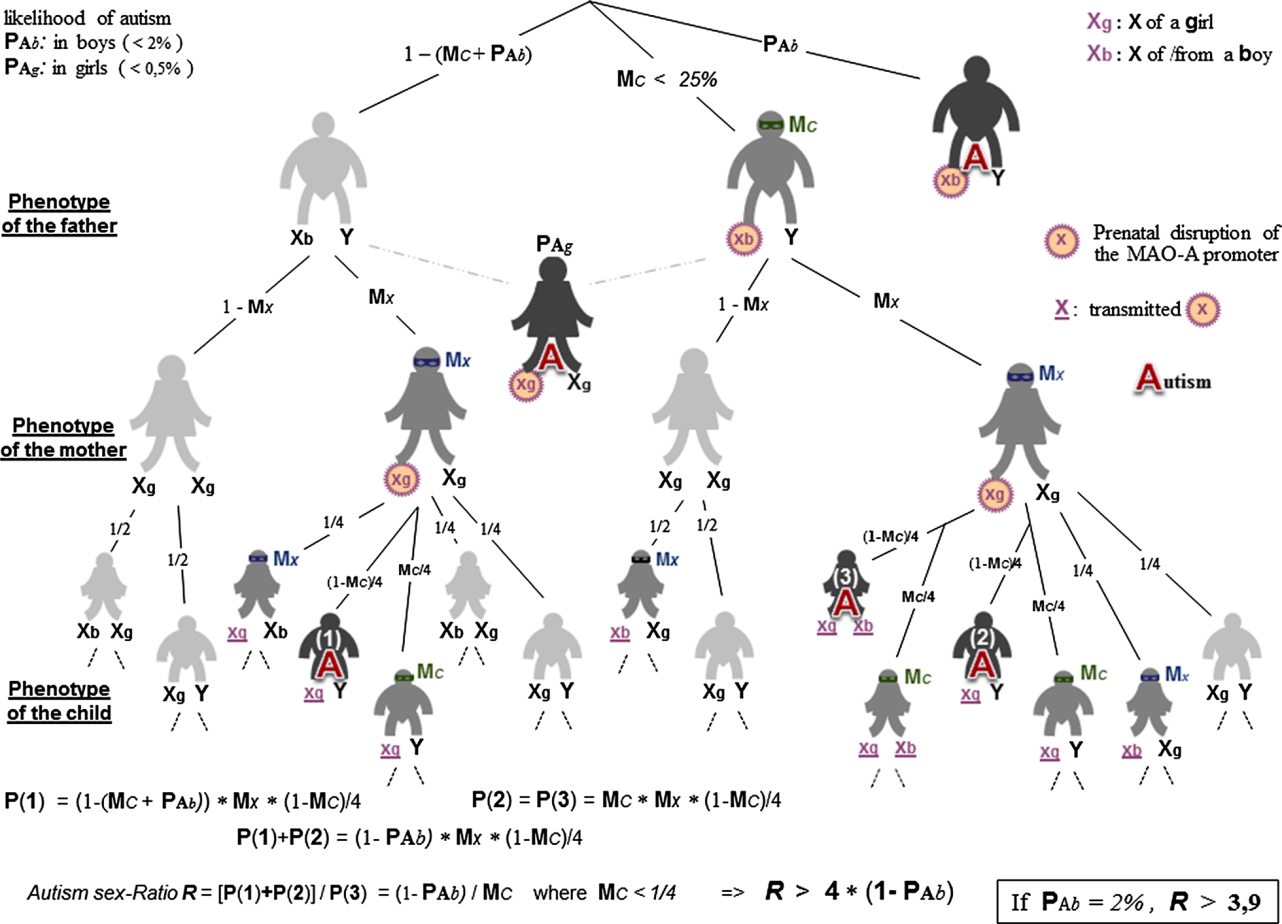
Family-tree displaying the phenotype of a child whose parents may convey a MAOA gene the down-promotion of which can be masked in two ways. Characters wearing a mask stand for people whose low-MAOA can be hidden by either neuromodulation or epigenetic masking. M_C_: In both genders, masking of a 5-HT noise (MAOA deficit) by a NE-noise (low-COMT allele). M_X_: In woman only, masking of a MAOA down-regulation in a single X-chromosome through the silencing process

The family tree of Fig. 9 shows how the genotypes of parents can combine to generate offspring with the male prevalence observed in ASD. According to this calculation, the autism sex-ratio would result from a possible congenital ‘genetic variant’ in both genders, together with an epigenetic protection of the only women, hence without deleterious genetic mutation. In these circumstances, both grandmothers of a woman with overt-autism would have been exposed to a harmful xenobiotic during their pregnancy, a relatively rare issue. Overt-autism would thus only constitute the tip of the iceberg that represents an entire generation, a situation that could underlie the current progression of the condition. A positive outcome however: if the primary structure of DNA is not altered [74], getting rid of deleterious environmental factors would gradually entail the disappearance of ASD together with its assumed epigenetic transmission.

## Discussion

### One parameter prone to encoding errors in different memory modules

The large amount of genes mutations already linked with autistic symptoms tends to reinforce the ongoing assumption of a multifactorial etiology. In contrast with this view, and independent of the actual distance between a model and its targeted biological processes, the computational results reported above show that a single parameter, if out-of-range, can generate aberrant representations that may underlie autistic features. The parameter in question (*R*) must decrease quickly enough to allow accurate memory encoding across ‘offline’ periods. Otherwise, disturbances arise in proportion to the clearing delay linked with the initial maximum value of *R* and its gradient (Fig. 3): the higher the remaining *R* when encoding is permitted in a given GP-module, the lower the ratio between well-formed and aberrant structures. Links converging from too many stimuli towards a given memory location can be considered as a source of perceptual over reactivity. In computer experiments, this convergence pattern has even generated cyclic out-of-control activations of GP-paths (not reported here). Links diverging from a single behavior-detector towards a chain of GP-cells within a deeper level code for repetitions of the same behavior, possibly irrelevant itself. These two connectivity patterns respectively support the perceptual hypersensitivity, evoke epilepsy crises, and stereotypy observed in ASD. A third discrepancy with the noise-free reference network lies in the greater amount of cells (× 1.6) and links that are brought into play, such as in the bigger brain of autistic persons [21]. As *w*_*5ht*_ is moved off its baseline “encoding value” in different computer sessions, the resulting networks exhibit more damaged modules, from those coding for emotional conditioning to those hosting sensorimotor representations. More precisely, a slight excess of *w*_*5ht*_ only impedes the ability to learn conditioned reflexes linked with relevant emotions. The associated “emotional channels” are also stimulated by larger receptive fields, because of convergent links. These symptoms recall the level-1 mild autism. With greater *R*, sensorimotor channels learn repetitions in fewer memory paths than in the reference network, hence similarities with the stereotypic and restricted behavior found in level-3 autism.

A spectrum of various behavioral deficits appears thus covered by incorrect values of a single operating parameter. Other factors may nevertheless modulate the expression of ASD-like symptoms. During simulated offline periods, the too-slow falling gradient of *R* can be offset by increasing the threshold at which encoding is possibly triggered (Fig. 3), which is a factor of structural variations in the resulting GP-network. A more biological source of variation is the timing of *cortical ontogenesis* in the fetal brain during pregnancy. During critical periods, specific populations of future neurons are migrating at different times from the *ventricular zone* towards different regions and layers of the cortex [75]. Depending on the time-interval of accidental monoaminergic imbalance (MAOA+, 5-HT-), the resulting long-term regulation would affect populations of cells destined for particular brain areas. If only acute during a short time-span, a xenobiotic intrusion may lead to epigenetic regulations of a restricted brain region, by contrast with the Brunner Syndrome genetic disease, in which the MAOA deficit concerns every cell of the body. Of note, this possibility questions the efficacy of drugs that feed the whole brain when only a few of its structures have been dysregulated. As shown in Fig. 1, changing the balance of monoamines could on the one hand improve affected neural circuits, while on the other hand dysregulate unaffected neurons. Back to the pathological architecture of sleep shown in Fig. 3, this point of the discussion also implies that the slope of the *R* gradient can be different from one GP-module to the other, which has not yet been tested.

### Possible causes of monoamines disruption in the fetal brain

Upstream a central cause of ASD proposed here, namely too-much persistency of 5-HT across sleep in a low-DA context, several causing factors can be put forward. In first place, the monoamine oxidase MAOA is the only enzyme which degrades 5-HT, and growing evidences from neurochemical, epidemiological and genetic studies support MAOA as one of the genes involved in autism. As mentioned above, the promotor region of this gene exhibits a functional polymorphism that reflects transcriptional activity variants. Since this built-in phenotype can also be found in non-autistic individuals, one is led to consider external events that are likely to interfere with the regulation of the MAOA promoter, instead. Indeed, a large number of environmental factors have been shown to modify the expression and activity of this enzyme [25]. The most aggressive ones are drugs which are known to increase enzymatic activity, such as *Forskolin* [76] notably used as food supplement for losing weight, and also VPA against epilepsy and bipolar disorders. According to the present theory, the same drug could induce opposite behavioral effects, whether mistakenly absorbed during pregnancy, or postnatally against established autism. This finding may shed light on the epigenetic management of environmental acute aggressions of gene promoters.

Following up with molecules at risk, two fatty acids have been identified in a systematic comparison of food ingredients and mood stabilizing drugs such as VPA. Computational results were confirmed in vitro within the same study [77]. Both molecules share at least an epigenetic influence with VPA, namely the *Histone Deacetylase* inhibition which increases the transcriptional activity of affected genes (through the under-compaction of *chromatin*). Although naturally occurring, their industrial counterparts are combined with additives in order to improve effectiveness, density and power of penetration into biological targets [78]. Extracted from pelargonium, *Nonanoic acid* is used as food preservative, among several others industrial applications. A broad-spectrum herbicide can also be obtained, through its combination with ammonia. Another molecule (*methyl nonanoate*) is composed of the same fatty acid and methanol, and used as flavors in food and perfumes industries [79]. *Decanoic* (or *caproic*) *acid* appears in both coconut and palm oils. Besides anti-seizure effect of this molecule (same medical effect as VPA), its various derivatives are manufactured as perfumes, lubricants, greases, rubber, dyes, plastics, food additives and pharmaceuticals [80].

Other combinations of atoms may share chemical, morphological and electrical properties with VPA, but most products of organic chemistry—including most drugs—cannot cross the *Blood*–*Brain Barrier* (BBB) because of either their molecular weight (> 400 g/mol) or their low lipid solubility (which is equivalent to high *hydrogen bounding*) [81]. However efficient this natural protection may be, including for the fetal brain, in vivo studies in rats indicate that exposure to low power, high frequency microwaves, both pulsed and continuous, affects the BBB permeability [82] which notably results in *albumin* leakage into the brain [83, 84]. A recent animal experiment confirmed these previous findings: 900 MHz electromagnetic field (EMF) radiations induce blood albumin into the brain, which is toxic to neuronal cells (the EMF power density near the head of the rats was adjusted to 1 mW/cm2 for 3 h per day for 28 days) [85]. The reported non-thermic effect is therefore likely to ease BBB crossing by fat-soluble pollutants possibly present in the blood, and the molecular weight of which is inferior to the albumin one. For instance, with its 158 g/mol and only 2 hydrogen bonds, the nonanoic acid is small enough to cross the BBB “by itself”, whereas the decanoic acid characteristics (408 g/mol and 7 hydrogen bonds) stand close to the limit values that forbid BBB crossing (400 g/mol and 8 hydrogen bonds [81]).

Trying to cope with the modern rise of brain development disorders, potential epigenetic disruptors of neurotransmitters are worth investigating without neglecting the EMF factor: not only particles/molecules but also waves/radiations might be involved.

### Involvement of structural and chemical factors

In computer science, malfunctions can be attributed either to hardware failures or to programming errors. This clear distinction between fixed hardware and changeable software hardly applies to neural networks, since they notably benefit from neurogenesis whereby they can evolve over a lifetime. Neuromodulation can however be considered as a software-like, dynamic element of the nervous system. When facing a pathology described by a variety of possible symptoms, it may be worth distinguishing between those involving slowly-varying neural structures from those reflecting dysregulations of dynamic neurochemical processes. For instance, treatments of some brain disorders (e.g.: Parkinson disease with drugs that increase DA levels) suggest that autism symptoms might as well be alleviated by neuromodulation changes. Indeed, stereotypies have been reduced by a 5-HT reuptake inhibitor (SSRI) in MAOA-KO mice [86], which questions the GP model. If, as central in this model, ASD originated in the poor clearance of 5-HT from the synapse, these symptoms should be exacerbated rather than alleviated by a drug which maintains synaptic 5-HT. However, the GP model implicates the offline processing stage, whereas effects of medications are usually considered in the waking subject. As previously noticed, the awake state brings its own way to deal with imbalanced neurotransmission. Thus, in the awake MAOA-KO mouse, a reduced 5HT neuronal activity can compensate for the lack of MAOA, perhaps at the expense of behavior. Indeed, a 40% reduction in spontaneous 5-HT firing is displayed by these mutants, a deficit known to be associated with a vulnerability factor for aggressive behavior [87]. Aggressive and impulsive behaviors that are observed in ASD could therefore be linked with a compensatory mechanism, namely a lower release of serotonin, itself possibly balanced by a SSRI. Despite the positive outcome of this target drug during the awake stage, the GP model predicts a worsening of long-term memory acquisition, especially if SSRI were evidenced to operate across sleep stages.

Another prediction of the GP model addresses the Brunner Syndrome issue, the human closest equivalent to the MAOA-KO mouse. Surprisingly, the former description of this genetic disease did not mention autism [15], which means that the full loss of MAOA function in every cell does not necessarily result in overt autism. Considered within the GP framework, this statement enhances the involvement of other factors, including the COMT upregulation (e.g.: through GG or Val/Val variant). Accordingly, only BS subjects with overactive COMT would exhibit autistic traits.

Back to the chemical or structural origin of ASD, the reduction of repetitive behavior by SSRI fosters a chemical impairment, whereas a structural defect is proposed by the GP approach. However, the last computer experiment reported above shows that a “GP hardware” in which faulty repetitions have been coded can be modulated afterwards so as to generate the proper sequence. This suggests that besides attenuating online behavioral disturbances, target drugs could mask an enduring structural deficit. By contrast, a disease-modifying treatment would rather prevent the neural hardware from subsequent memory impairments. Uncovering in this way the nature of ASD symptoms would help to orientate therapeutic strategies. In any case, because the time course of therapeutic effects may take weeks or even months after an immediate chemical modification, it seems that potential medications should be conducted long enough to assess their actual impact on learning and memory. Different molecules may be required depending on targeted states, either awake or sleep, in the hope that they complement each other, or at least do not show antagonist effects over the same stages of brain function.

### Postnatal outset and possible reduction of autistic symptoms

The ASD pre-natal origin assumed here is questioned by the behavioral regression that often initiates overt-autism months after a regular post-natal development [32]. At the beginning of pregnancy, the critical period of neurulation is characterized by the proliferation of neural stem cells, as well as by the epigenetic programming of genes expression (*methylation*) patterns that are transmitted to generations of neurons throughout life [87]. These epigenetic mechanisms are likely to maintain homeostasis (e.g.: 5HT-NE coupling, accessible through cellular feedback) by counteracting the effect of a xenobiotic. Whereas the initial pool of neurons and glia cells may benefit from this epigenetic balancing, the following cells generations would only undergo the long-term relevant regulation after the end of the ‘excess MAOA’ episode [27] (Fig. 1). Before the 2-year delay for MAOB to significantly impact DA and presumably uncovers the 5HT-NE uncoupling, the latter would arise with the exhaustion of the first pools of neural stem cells for which the persistent regulation was initiated. According to the study presented here, a life-long treatment targeting the metabolism of monoamines should then be initiated as soon as possible to prevent aberrant structures that are assumed to underlie ASD core symptoms.

In order to prepare relevant clinical trials, a preliminary case-study has been conducted over one year, involving an 11-year old boy with severe autism [88]. This disease-modifying treatment, associated with a timely progressive schooling, is based on two complementary drugs aimed at rebalancing the metabolism of monoamines (on the right side of Fig. 1). Specific biomarkers are propounded for monitoring future trials in which every trait of the condition should be quantified in various participants over more than 10 months. These biomarkers involve monoamines and their enzymes variants (MAOA/B, COMT), plasma VPA levels, sleep-EEG, and eye-tracking.

## Conclusion

The theory introduced in this article suggests that ASD originate in environmental factors modulated—or even hidden—by genetic variants. The current rise of this condition would be favored by three sources of biological masking: (1) during sleep, mostly overnight, when the cyclic variations of monoamines are assumed to undergo disrupted enzymatic activities; (2) through generations of ‘healthy carriers’ protected by the X-chromosome silencing and the low-COMT phenotype, consistently with the autism sex-ratio; (3) early in life, as long as the brain development draws on pools of neurons born when the prenatal accident and its epigenetic regulation overlapped, and until MAOB starts contributing significantly to the synaptic degradation of dopamine. Once the causing prenatal accident is over, its persistent regulation would impair sleep and the brain development throughout life.

Therefore, overt-autism would be:

- caused by the persistence of epigenetic regulations (MAOA-, COMT +) after the end of a prenatal monoaminergic disruption (excess MAOA). Relevant epigenetic traits might be:
  - transmitted to progeny,
  - maintained across generations through causal environmental factors,
- masked in “healthy carrier” parents by epigenetic and neuromodulation mechanisms,
- modulated on the one hand by the period of prenatal accident, and, on the other hand, by inherited polymorphisms of genes involved in the management of monoamines.

The study that led to this scenario combines in the same framework features issued from different science subjects, with status ranging from established facts to new hypotheses. For example, while the environmental susceptibility of genes promoters is widely acknowledged, the inheritance of their epigenetic programming is still questioned in humans. Among other hypotheses that were previously formulated, the coupling of monoamines is brought here for an interpretation of the autism sex-ratio, provided a masking effect propagated across human generations. Although neurogenesis cannot be denied anymore, the possibility that successive pools of new neurons are aimed at growing inner representations of events experienced all along the lifespan is not quite recognized. Combined here with early epigenetic reactions to a monoaminergic disruption, a continuous neurogenesis allows an initial pool of neurons to be replaced by waves of new cells. Whereas 5-HT levels would be balanced in neurons which are born while the dysregulation and its epigenetic response co-occurred, no later than the end of gestation, the following generations of neurons would only undergo the obsolete epigenetic regulation. A post-natal regression would then gradually emerge, enhanced after the time at which MAOB reaches a high in its activity. Another new assumption even questions the current multifactorial view on the origins of the ASD heterogeneity: here, only two enzymatic variations acquired before birth would cover a spectrum of brain developmental disorders, as a function of the intensity of these variations, their phenotypic context, and the prenatal period at which the causing accident occurred. No primary genetic mutation would be involved as a key factor of the condition. Across the “roots of autism” status-bar that goes from “genetic” to “environmental”, the conclusion drawn here therefore contributes to set the slider close to an environmental etiology. Regarding a new hypothesis that deserves closer examination, sleep is paralleled with the ‘offline’ encoding period required by the GP model.

This research can be pursued in both theoretical and experimental directions. An investigation should be conducted in order to identify the environmental factors suspected to trigger specific epigenetic regulations in the fetal brain: the ones that may turn out to underlie autistic symptoms. These factors include xenobiotics, some of which are already known. Depending on both its intensity and various contexts in which it may occur, the involved monoaminergic imbalance is assumed here to result in various cases across the spectrum of autistic disorders. Based on this preliminary outline, a more exact picture remains to be drawn. As now recognized, intellectual disabilities associated with ASD are notably modulated by the background phenotype, namely the allelic variants of gene promoters involved in the monoamines transport and metabolism. Given the interaction between neurotransmitters that control the brain functions, all of them should be considered for mapping genetic variants onto the severity of impairments. If enzymes are programmed in neural structures when they are initiated in the fetal brain, the period at which a xenobiotic occurs would determine what brain circuits consequently undergo developmental deficits. The numerous clinical manifestations of autism would be traced to as many possible combinations of the fetal genotype relating neurotransmitters with the prenatal period of adverse exposure. As highlighted by the computational model proposed here, the intensity of the *w*_5ht_-noise parameter can also be combined with an assumed threshold at which memory encoding is triggered. These two parameters should be considered separately in future computer experiments, for assessing their respective involvement in simulated ASD. As these parameters are shifted from their baseline range, specific disruptions of the offline processing could form activity patterns to be compared to those found in sleep-EEGs. In the best-case scenario, the detection of these abnormal patterns may help to predict the severity of autistic disorders.

In addition to the aforementioned potential masks of autism symptoms, a GP-model trial showed that a structural defect (i.e.: aberrant connectivity) could be hidden by an effective modulation of parameters (i.e.: withdrawal of time delays) involved in the functioning of these structures. In neurobiological words, this result suggests that psychotropic drugs could overcome a behavioral deficit without modifying its structural basis. The medical approach promoted here is rather to target the earliest possible rebalancing of neuromodulation, if eventually held responsible for long-term structural impairments underlying ASD. Beside computer experiments, clinical trials may take advantage of the preliminary case-study driven by this study. Hopefully carried out in both children and adults for at least one year, related future trials should rely on specific biomarkers analyzed before and across a treatment based on regulators of the monoamines metabolism, and associated with proper rehabilitation programs. Taking into consideration the complexity of the metabolic pathways involved in autism, the underlying model is likely to be refined and completed in parallel.

## Methods

The software implemented in this study is derived from the GP model of emotional decision-making [66]. Similarly, the input patterns of activity represent behaviors, while the software output consists of several types of information delivered over a computer session: labels of memory paths whenever they are created, amount of cells and links within/between modules, and activity histograms of selected memory paths.

### System input

The alternation of stimuli and actions in GP memory paths was initially proposed in the context of trial-and-error problem solving [89]. In the present system, a given input involves sequences of stimuli, either sensory or *proprioceptive* (from ongoing actions) that are dispatched to parallel GP channels, with their respective internal representations. The input data is composed of symbols: consonants, vowels, and digits that respectively code for sensory stimuli (including 6 unconditional ones: *UCS*), proprioceptive stimuli, and emotional inner-states. The input data is contained in a ‘behaviors’ file (Additional file 1), each line of which is a sequence of digits and 3-letter words from the French *Scrabble* in the same format as the following instance: 0BUS 2KYT 1LOC., where 0 is the “neutral emotion” inner-state, B and U are neutral stimuli, and S is a ‘conditioner’ stimulus with a prewired emotional valence. This valence results in the change of inner-state represented by digit 2, and so on. Spaces stand for boundaries between elementary behaviors, and help the learning algorithm to build its hierarchy of modules: references of elementary behaviors (e.g.: BUS) will appear in the most peripheral layer, while their combinations will be represented in the second layer.

8 banks of ED/Gs stand at the input of the GP channels. Whereas *Hippo* is fed by the full input (nested series of elementary patterns: *State*-*Stim*-*Action*-*UCS*), other channels are more specialized: channel 1 encodes the conditioning of simulated ‘inner-states’ (*State* =>*UCS*), and selectively facilitates channel 2 where neutral stimuli are conditioned (*Stim* =>*UCS*), which itself either facilitates or repress the sensorimotor patterns of channel 3: *Stim* =>*Action* =>*UCS*.

### Transformations

**Fig. 10:**
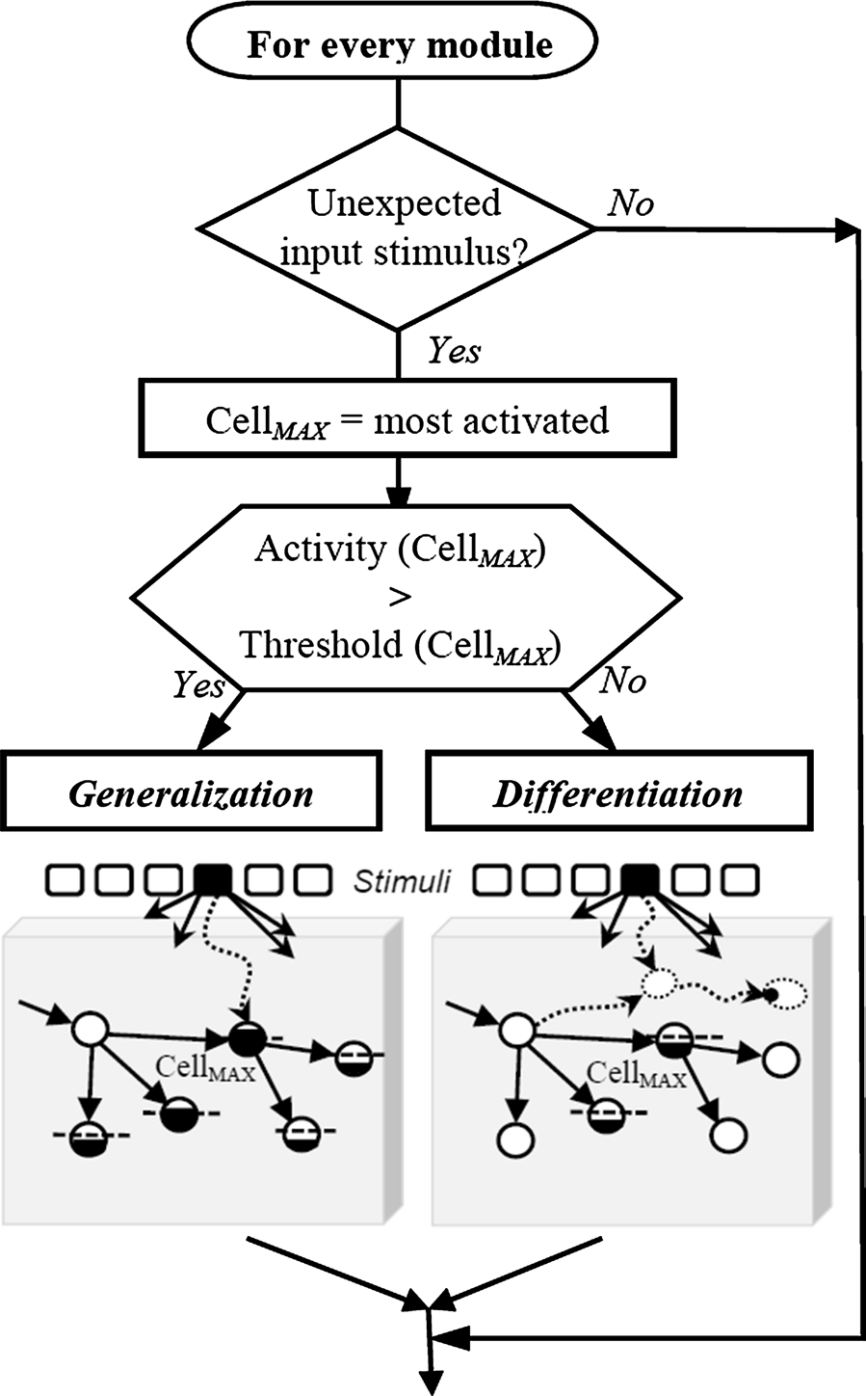
(From [51]). Growth of a GP module, based on the coincidence between an inner-flow and incoming stimuli. Alternative encodings of a given stimulus by a downstream module are shown in the two grey boxes at the bottom; the choice between two patterns of growth (dotted arrows) depends on the level of activity of coincident cells, itself controlled by the current weight *w*_5ht_ of the inner-flow. Here, the filling level of a cell stands for its activity, whereas a dashed-line features its response threshold. Thanks to its tree-like structure that results from several encoding steps, a given GP module can anticipate the next stimuli to occur. If a stimulus occurs which was not expected in the ongoing context, the learning algorithm is triggered. The more boosted the inner-flow then, the more *Generalization* takes the lead over *Differentiation*

At the system initiation, every module only contains a root cell, from which memory-paths expand according to the Differentiation/Generalization algorithm (Fig. 10).

**Fig. 11:**
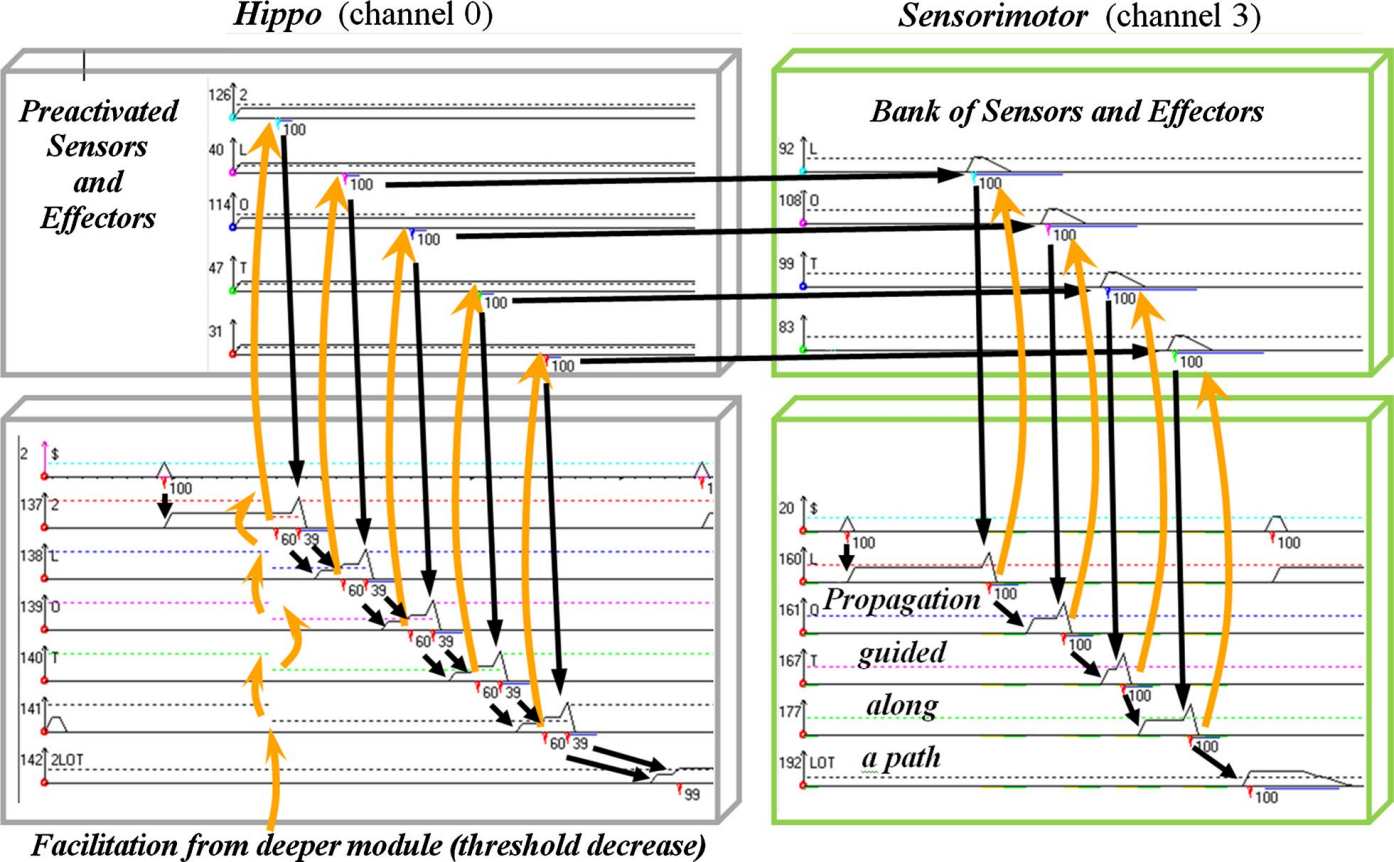
Offline, GP-cells histograms displayed along the replay of a pattern by *Hippo*. Activation (black arrows) of a sensorimotor path (on the right side) occurs through the facilitation (upward orange arrows) of its correspondent/partner path of *Hippo*, via connected banks of sensors/effectors (peripheral ED/Gs). In the left-top module, the *Hippo* sensors/effectors must be pre-activated below their decision thresholds for a facilitation signal to trigger their response

Compared with previous GP software, this computer simulation mainly involves a supplementary channel (i.e.: *Hippo*) aimed at the online activation/recognition of memory paths representing known behaviors in a global format, but also, and particularly, for the fast recording of unexpected sequences of stimuli. Across the following ‘offline’ stage, related memory paths are repeatedly activated through modulation signals, with the effect of replaying the same behavioral patterns as ‘online’ (see Fig. 11). Other GP modules can thus be stimulated by *Hippo* productions via pre-activated sensors and effectors (ED/G cells). Among them, a behavior not yet represented in a given module can trigger an encoding session within this module, provided that the following constraints are satisfied:

- At least one free GP-cell is available.
- During the ongoing ‘offline’ cycle, the encoding threshold has been reached by the decreasing variable *R* associated with the module cells (Figs. 3, 4).
- The *Control Unit* (i.e.: software routine) allows the module extension, based on its current connectivity with *Hippo*. The goal is to properly rank encoding across a hierarchy of layers and parallel channels that may share the same input. For this purpose, the ED/G output of every new path is connected through a loop towards its correspondent in *Hippo*, namely the ED/G output of its initiator memory path(s).
  - The *n*th layer of a given channel is allowed to encode a new pattern as soon as its elementary components (of layer *n* − *1*) have all previously been learnt. This happens when a path of the *n*th *Hippo* layer is being activated by ED/G that all received a loop connection.
  - Cross-circuits can only start growing when *Hippo* has received feedback loops from its three correspondents of other channels.

### System output

The comparison between the networks respectively issued from different parametrizations is based on the labels of their respective ED/G cells (see Table 3).

**Table 3:**
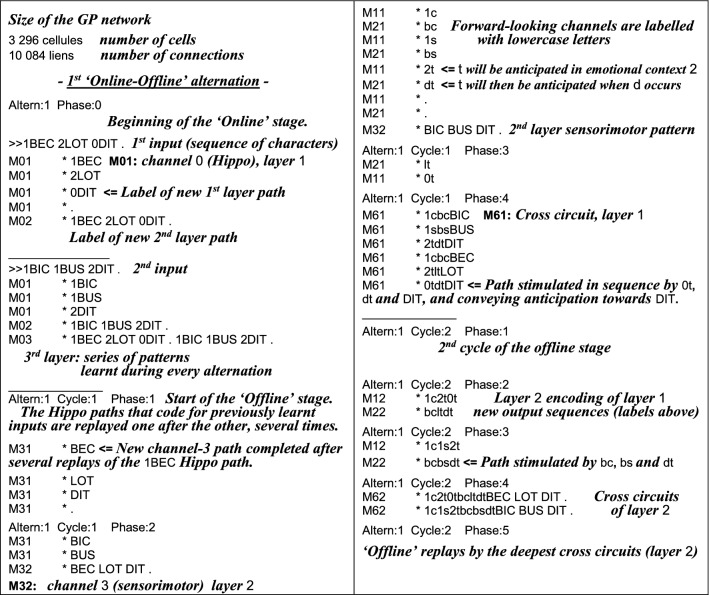
Excerpt from an output file containing records of the labels assigned by the computer simulation to newly encoded memory paths

Added explanatory comments appear in bold italics

For a closer look to the system local behavior, histograms show the activity spreading along selected memory paths while the software is running (Fig. 11). The global activity across time, counters of cells and links are also recorded for each module. Given the deterministic nature of the GP model, no statistical tool is required when the input data stays under the user’s control and does not generate uncertainty.

### Experiments

When a trial begins, every module only contains a root cell which is the point at which the first memory-paths start growing. Distant connections between the outputs of *Hippo* and other channels, as well as the Control Unit are aimed at managing online/offline alternations. The symbolic inputs/outputs standing for compound behaviors are eventually represented within two layers and four channels of GP-network.

The networks that are built from input patterns can be item-per-item compared to each other, thanks to the deterministic nature of this approach. More precisely, a reference network is first built from a corpus of simulated behaviors with proper values of GP-cell parameters (*R* = *w*_*5ht*_*/w*_*ne*_ and *Excitability* in Fig. 4). Then, each value of *w*_5ht_ noise generates its own network from the same input data, to be compared to the reference network. The main experiments of this study dealt with 50 online/offline alternations during which the system was fed with 64 combinations of 100 elementary patterns. The offline encoding was assessed by checking how many paths were either preserved or defective (i.e.: with an aberrant label) for noise levels ranging from *1.1* to *2.5* (divider of the *R* noise-free gradient).

The processing of 100 input samples (two ‘behaviors’ per alternation, 50 online/offline alternations) which leads to the baseline (noise-free) network currently uses up to 300 Mo of RAM and takes 30 min on a computer with a 3.30 GHz CPU.

## Additional file

**Additional file 1.** Data used in the reported experiments. Full set of symbolic input representing behaviors, with which a given GP computer simulation can be fed across a series of 50 simulated online/offline alternations. The GP network grows from scratch when an unexpected behavior occurs in its input. A reference network is first obtained with baseline control parameters, and can be compared with other instances grown when parameters are shifted in a particular way (e.g.: according to the GP model of autism).

## Abbreviations

ASD: autism spectrum disorders
5-HT: serotonin
BBB: blood–brain barrier
BS: Brunner syndrome
COMT: catechol-O-methyltransferase
DA: dopamine
DNA: deoxyribonucleic acid
ED/G: event detector/generator
EMF: electromagnetic field
GP: guided propagation
IME: Educational and Medical Institute
MAO: monoamine oxidase (either type A or B)
MDD: major depressive disorder
NE: norepinephrine/noradrenaline
US: unconditioned stimuli
REM: rapid eye movement
SSRI: selective-serotonin reuptake inhibitor
SWS: slow-wave-sleep
VPA: valproate, valproic acid
