# Supplementary material for: Offline encoding impaired by epigenetic regulations of monoamines in the guided propagation model of autism
Source: BMC Neurosci. 2018 Dec 17;19:80. doi: 10.1186/s12868-018-0477-1 (PMC6298000; doi:10.1186/s12868-018-0477-1)
Supplement: Supplementary file 1 — Additional file 1. Data used in the reported experiments. Full set of symbolic input representing behaviors, with which a given GP computer simulation can be fed across a series of 50 simulated online/offline alternations. The GP network grows from scratch when an unexpected behavior occurs in its input. A reference network is first obtained with baseline control parameters, and can be compared with other instances grown when parameters are shifted in a particular way (e.g.: according to the GP model of autism). [file 12868_2018_477_MOESM1_ESM.pdf]

Additional file 1

Data used in the reported experiments

Each temporal pattern of conditioning:

Inner state – Stimulus - Proprioception – Uncontioned Stimulus

...is formatted as follows:

Digit [0, 4] - Consonant – Vowel - Consonant among a preset

The GP-network is fed with the following combinations of 3 elementary patterns formatted as above.

Alternation **1**
1BEC 2LOT 0DIT .
1BIC 1BUS 2DIT .

Alternation **2**
4BUT 0DES 3WOS .
1MAC 2FOC 2BAS .

Alternation **3**
2LOT 0MAC 0DIT .
0BUS 2KYT 1LOQ .

Alternation **4**
4LOT 3BUT 3DES .
1LOT 3KIS 4LAS .

Alternation **5**
0BIS 4ROT 3FAC .
4DIT 3BON 3DES .

Alternation **6**
2DAN 1FAC .
1LOT 3KIS 4LAS .

Alternation **7**
0BIS 4ROT 3FAC .
3DES 4GAT 3BON .

Alternation **8**
0DES 0HIC 1JAN .
1MAC 2FOC 2BAS .

Alternation **9**
3MUS 4MOT 0PUC .
0VIS 1PEC 2KAT .

Alternation **10**
1DOT 3PAS 4MAC .
3JAN 3LAS 0ROT .

Alternation **11**
0MAT 1RUT 1WON .
1BIC 1BUS 2DIT .

Alternation **12**
3HES 3PUS 4MOT .
4MOT 3POT 3PIN .

Alternation **13**
2KAN 2MON 1PAS .
0MAX 4MIN 1HOT .

Alternation **14**
1ROC 2FAC 2FIN .
3PYS 4DYT 3JYS .

Alternation **15**
0NEZ 5JET 3NYS .
0MAC 2DOT 3PAS .

Alternation **16**
0PUS 4RUZ 5KAT .
0ROT 3JAN 1LAS .

Alternation **17**
0RAZ 5KOT 3PAS .
0WON 1MAT 3RUT .

Alternation **18**
0KYT 3PYS 4RAZ .
0DIT 3BIC 2BUS .

Alternation **19**
0REZ 5LET 3RIS .
0HES 4PUS 4MOT .

Alternation **20**
0BON 1RAS 4LYT .
0PIN 1MOT 3POT .

Alternation **21**
0BAN 1LOT 3RYS .
0PAS 4KAN 1MON .

Alternation **22**
0VOS 4BEN 1MIT .
0HOT 3MAX 6MIN .

Alternation **23**
0CIN 1MOT 3VAS .
0FIN 1ROC 2FAC .

Alternation **24**
0MAT 3VIS 4DON .
0JYS 4PYS 4DYT .

Alternation **25**
0DAN 1NAT 3VUS .
0MAC 2DOT 3PAS .

Alternation **26**
0DIN 1WOS 4NUT .
0ROT 3JAN 1LAS .

Alternation **27**
0FAN 1NIT 3CUX .
0WON 1MAT 3RUT .

Alternation **28**
0FIN 1PIT 3BEN .
0DIT 3BIC 2BUS .

Alternation **29**
0GIN 1PET 3FAX .
0HES 4PUS 4MOT .

Alternation **30**
0HUN 1POT 3DON .
0PIN 1MOT 3POT .

Alternation **31**
0HAN 1RUT 3GEX .
0PAS 4KAN 1MON .

Alternation **32**
0JAN 1RET 3NUT .
0HOT 3MAX 6MIN .

Alternation **33**
0KIN 1ROT 3DOT .
0FIN 1ROC 2FAC .

Alternation **34**
0KAN 1VET 3MIT .
0JYS 4PYS 4DYT .

Alternation **35**
0LON 1VYT 3MAX .
0MAC 2DOT 3PAS .

Alternation **36**
0MIN 1BIS 4KAT .
0ROT 3JAN 1LAS .

Alternation **37**
0MIN 1BUS 4FEZ .
0WON 1MAT 3RUT .

Alternation **38**
0MON 1BAS 4GET .
0DIT 3BIC 2BUS .

Alternation **39**
0NON 1CAS 4JUT .
0PIN 1COS 4CET .

Alternation **40**
0PAN 1CIS 4RUZ .
1BEC 2LOT 0DIT .

Alternation **41**
0QYN 1DES 4NUT .
1BIC 1BUS 2DIT .

Alternation **42**
0VON 1DOS 4RAZ .
4BUT 0DES 3WOS .

Alternation **43**
0VAN 1DUS 4DON .
1MAC 2FOC 2BAS .

Alternation **44**
0WON 1FYS 4LYT .
2LOT 0MAC 0DIT .

Alternation **45**
0CUX 6FAN 1NIT .
0BUS 2KYT 1LOQ .

Alternation **46**
0BEN 1FIN 1PIT .
4LOT 3BUT 3DES .

Alternation **47**
1LOT 3KIS 4LAS .
0BIS 4ROT 3FAC .

Alternation **48**
3DES 4GAT 3BON .
0DES 0HIC 1JAN .

Alternation **49**
1MAC 2FOC 2BAS .
3MUS 4MOT 0PUC .

Alternation **50**
0VIS 1PEC 2KAT .
1DOT 3PAS 4MAC
